# Micron-sized single-crystal cathodes for sodium-ion batteries

**DOI:** 10.1016/j.isci.2022.104205

**Published:** 2022-04-04

**Authors:** Venkat Pamidi, Shivam Trivedi, Santosh Behara, Maximilian Fichtner, M. Anji Reddy

**Affiliations:** 1 Helmholtz Institute Ulm (HIU) Electrochemical Energy Storage, Helmholtzstraße 11, 89081 Ulm, Germany; 2 Institute of Nanotechnology, Karlsruhe Institute of Technology, PO. Box 3640, 76021 Karlsruhe, Germany; 3 Faculty of Science and Engineering, Swansea University, Fabian Way, Swansea SA1 8EN, UK

**Keywords:** Electrochemistry, Energy systems, Materials science

## Abstract

Confining the particle-electrolyte interactions to the particle surface in electrode materials is vital to develop sustainable and safe batteries. Micron-sized single-crystal particles offer such opportunities. Owing to the reduced surface area and grain boundary-free core, particle-electrolyte interactions in micron-sized single-crystal particles will be confined to the particle surface. Here, we reveal the potential of such materials in sodium-ion batteries. We synthesized and investigated the chemical, electrochemical, and thermal properties of single-crystalline P2-type Na_0.7_Mn_0.9_Mg_0.1_O_2_ as a cathode material for sodium-ion batteries. Single-crystalline Na_0.7_Mn_0.9_Mg_0.1_O_2_ with a mean particle size of 8.1 μm exhibited high cycling and voltage stability. In addition, the exothermic heat released by the charged single-crystal Na_0.7_Mn_0.9_Mg_0.1_O_2_ cathodes was four times lower than that of the corresponding polycrystalline Na_0.7_Mn_0.9_Mg_0.1_O_2_. This significantly enhances the thermal stability of electrode materials and possibly mitigates thermal runaways in batteries. Surprisingly, single crystals of Na_0.7_Mn_0.9_Mg_0.1_O_2_ were relatively stable in water and ambient atmosphere.

## Highlights

• Micron-sized single crystals (MSSC) of P_2_-Na_0.7_Mn_0.9_Mg_0.1_O_2_ were investigated for SIBs
• They showed high cycling and voltage stability compared to the polycrystalline sample
• Exhibited four times lower exothermic heat evolution than the polycrystalline sample
• MSSC cathode showed higher stability in air and water

## Graphical abstract

**Figure:**
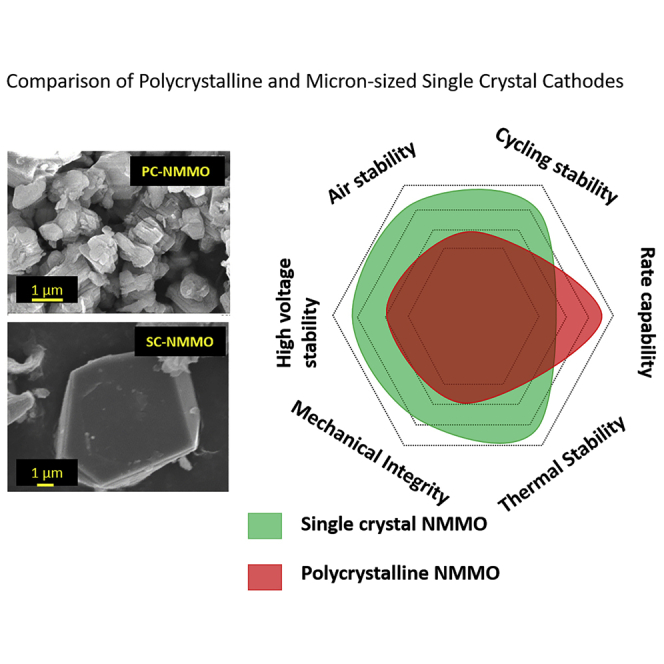

## Introduction

Sodium-ion batteries (SIBs) have a high potential to substitute/complement current lithium-ion batteries (LIBs). SIBs are particularly suitable for power tools, grid energy storage, and heavy-duty electric vehicle applications considering their fast charge/recharge capabilities, moderate energy density, sustainable resources, and predicted low cost (Tapia-ruiz et al., 2021; Vaalma et al., 2018; Yabuuchi et al., 2014c). However, several fundamental and technological issues need to be addressed to realize the full potential of SIBs. While hard carbon turned out to be a suitable anode, a high-energy, long-life, and safe cathode material is yet to be realized for SIBs. Polyanion-based Na_3_V_2_(PO_4_)_3_ and related materials offer high cycling stability. However, their energy density is low and capped, the material is relatively expensive, and large-scale synthesis will be cumbersome (Barpanda et al., 2018; Senthilkumar et al., 2019). On the other hand, similar to LIBs, layered cathode materials are attractive for SIBs due to their high specific capacity, mixed conductivity (both electronic and ionic), ease of synthesis on a large scale, and potentially low cost. On the downside, during Na removal/reinsertion, layered cathode materials undergo large volume changes and reversible/irreversible phase transformations (depending on the structure type and charging voltage limit). This eventually leads to poor cycling and low thermal stability (Goikolea et al., 2020; Han et al., 2015; Liu et al., 2019; Ortiz-Vitoriano et al., 2017; Song and Kendrick, 2021; Xiao et al., 2021). Unlike in LIBs, some of the layered oxide cathode materials in SIBs suffer from oxygen redox (again depending on the structure type, composition, and charging voltage limit), which can trigger oxygen release in the charged state, aggravate the parasitic reactions, and result in lower thermal stability (Kang et al., 2020; Maitra et al., 2018; Rahman and Lin, 2021). Further, Na_x_MO_2_-type layered oxides show low chemical stability in the ambient atmosphere (Zuo et al., 2020). These critical issues associated with layered cathode materials of SIBs need to be addressed before the door for commercial applications can be opened. Indeed, numerous layered cathode materials with different elemental compositions have been investigated to enhance the cycling stability of layered cathode materials for SIBs (Chen et al., 2018; De La Llave et al., 2016; Lu and Dahn, 2001; Talaie et al., 2015; Yabuuchi et al., 2012a, 2014b; Zhao et al., 2014). Further, surface coatings have been suggested to improve the cycling and air stability of layered cathodes (Hwang et al., 2017; Jo et al., 2018; Yu et al., 2015).

Recently, micron-sized single-crystal (MSSC) cathodes have gained a lot of attention for LIBs (Fan et al., 2020; Langdon and Manthiram, 2021; Li et al., 2017; Qian et al., 2020; Wang et al., 2021). Fan et al. investigated the micron-sized single crystals of Ni-rich LiNi_0.83_Co_0.11_Mn_0.06_O_2_ (SC-NCM), which exhibited significantly improved cycling stability at room temperature as well as at 55°C compared to polycrystalline materials. Additionally, pouch-type full cells of SiO-C/SC-NCM delivered capacity retention of 84.8% after 600 cycles at a rate of 1C at 45 °C (Fan et al., 2020). Li et al. achieved excellent capacity retention of ∼92% even after 1600 cycles at C/2 at 40°C for single crystal LiNi_0.5_Mn_0.3_Co_0.2_O_2_. Further, through thermogravimetry and mass spectroscopy analysis, they revealed that the single crystal LiNi_0.5_Mn_0.3_Co_0.2_O_2_ exhibited higher resistance to oxygen loss compared to the polycrystalline material (Li et al., 2017).

A single crystal is a particle without a grain boundary in its bulk. Usually, particles without grain boundary can be called single crystals irrespective of size. However, the single-crystal cathodes we are interested in and refer to in this work are micron-sized particles in a size range of 1–10 μm. Owing to the low surface area and grain boundary-free core, the interactions between particle and electrolyte in micron-sized single-crystal particles will be reduced significantly compared to polycrystalline particles. Furthermore, as the particle and electrolyte interactions are confined to the particle’s surface, the charge transfer and subsequent ion diffusion in the bulk of the particle can be viewed as quasi-solid-state processes in single-crystalline particles. Consequently, a series of advanced properties can be expected from MSSC cathodes. These materials consistently outperformed the polycrystalline (PC) equivalents in LIBs. MSSC cathodes showed high cycling stability (at RT and elevated temperatures), high voltage stability, reduced parasitic side reactions between cathode and electrolyte, reduced degree of microcracking, and enhanced thermal stability (Chiba et al., 2020; Jiang et al., 2021; Klein et al., 2021; Li et al., 2021b; Qian et al., 2020).

Inspired by the great advantages offered by microscale single crystals in LIBs, we extended the concept of MSSC cathode to SIBs. We have chosen P2-type Na_0.7_Mn_0.9_Mg_0.1_O_2_ (NMMO) for our studies, as it is composed of sustainable elements, free of Co and Ni, and has proven to be a cathode material for SIBs with high capacity (Billaud et al., 2014; Yabuuchi et al., 2014b). In this study, we have synthesized and investigated the electrochemical properties of single-crystalline P2-type Na_0.7_Mn_0.9_Mg_0.1_O_2_ with various particle sizes for SIBs for the first time. Briefly, we synthesized NMMO with various particle sizes and investigated their structure, morphology, chemical stability, electrochemical performance, thermal stability of charged cathodes, and finally, the capacity degradation mechanisms of NMMO were studied.

## Results and discussion

### Phase stability and chemical compatibility of NMMO

Polycrystalline Na_0.7_Mn_0.9_Mg_0.1_O_2_ was synthesized by mechanically milling stoichiometric amounts of Na_2_CO_3_, MnO_2_, and MgO for 2 h at 500 rpm followed by calcining this mixture at 900°C for 12 h in air (NMMO-900). NMMO-900 was then heated in different batches at 1000°C, 1050°C, 1100°C, and 1150°C for 12 h in air and denoted hereafter as NMMO-1000, NMMO-1050, NMMO-1100, and NMMO-1150, respectively. No excess Na_2_CO_3_ was used for samples calcined at 900°C, 1000°C, and 1050°C. Nevertheless, phase-pure NMMO-1100 and NMMO-1150 were obtained only after adding 10% and 20% excess Na_2_CO_3_, respectively, which is necessary to compensate for the Na loss at these temperatures. Impure phases were obtained when NMMO-900 was calcined at 1200°C and 1300°C even with 50% excess of Na_2_CO_3_ (Figure S2). A detailed synthesis procedure is given in the experimental section.

**Figure 1:**
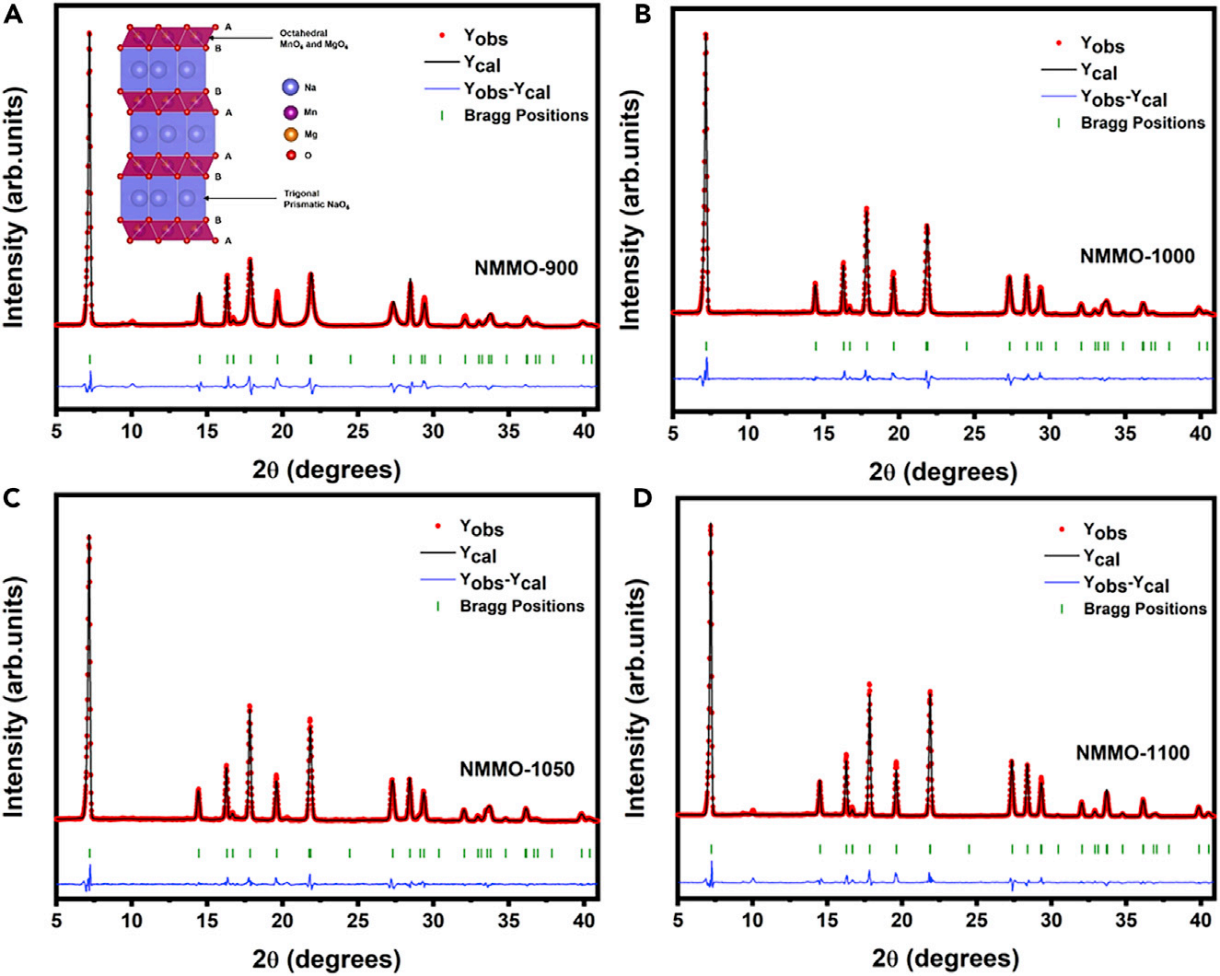
Rietveld-refined powder X-ray diffraction patterns of NMMO cathode materials synthesized at different temperatures (A–D) (A) 900°C, (B) 1000°C, (C) 1050°C, and (D) 1100°C. Dots represent observed data, the solid line represents a calculated pattern, and the lower line is the difference between them. Bragg positions are shown as vertical lines. Rietveld refinement was performed using FullProf software. The inset in (A) is a schematic illustration of the P2-NMMO structure drawn using VESTA software. See also Figures S1–S3.

Figure 1 shows Rietveld-refined X-ray diffraction (XRD) patterns of NMMO calcined at different temperatures for 12 h (900°C–1100°C). All NMMO samples synthesized could be indexed/fitted well with the parent Na_0.7_MnO_2.05_ pattern with a layered hexagonal structure and a space group of P6_3_/mmc (ICSD file: 00-027-0751). The corresponding crystallographic data, calculated lattice parameters, and occupancies are presented in Figure S1 and Table S1. A marginal peak shift to lower angles was observed for NMMO due to the substitution of larger size Mg^2+^ in place of Mn^3+^/Mn^4+^ (Figure S1A). Further, the (002) planes at 2θ = 7.19° shifted to lower angles in NMMO-1000 and NMMO-1050 compared to NMMO-900 (the expanded figure is shown in Figure S1B), indicating partial loss of Na. The loss of Na would increase the distance between the MO_2_ layers as the electrostatic repulsion between the negatively charged MO_2_ layers increases. The lattice parameters of all NMMO samples are plotted and shown in Figure S1C). The c lattice parameter increased from NMMO-900 to NMMO-1050 but slightly decreased in NMMO-1100 and NMMO-1150. This slight decrease of c in NMMO-1100 and NMMO-1150 could be due to the partial compensation of Na loss due to the excess use of Na_2_CO_3_. Both, the lattice parameter and unit cell volume are gradually increased from NMMO-900 to NMMO-1150. The composition of as-prepared NMMO-900 and NMMO-1100 compounds was Na_0.61_Mn_0.84_Mg_0.09_O_2_ and Na_0.57_Mn_0.83_Mg_0.09_O_2_, respectively, which is evaluated by inductively coupled plasma-optical emission spectrometry (ICP-OES). The estimated composition was close to the stoichiometric composition. The surface area of NMMO-900 and NMMO-1100 was estimated to be 1.98 and 0.68 m^2^/g. Nitrogen adsorption/desorption isotherms of NMMO-900 and NMMO-1100 are shown in Figure S3.

**Figure 2:**
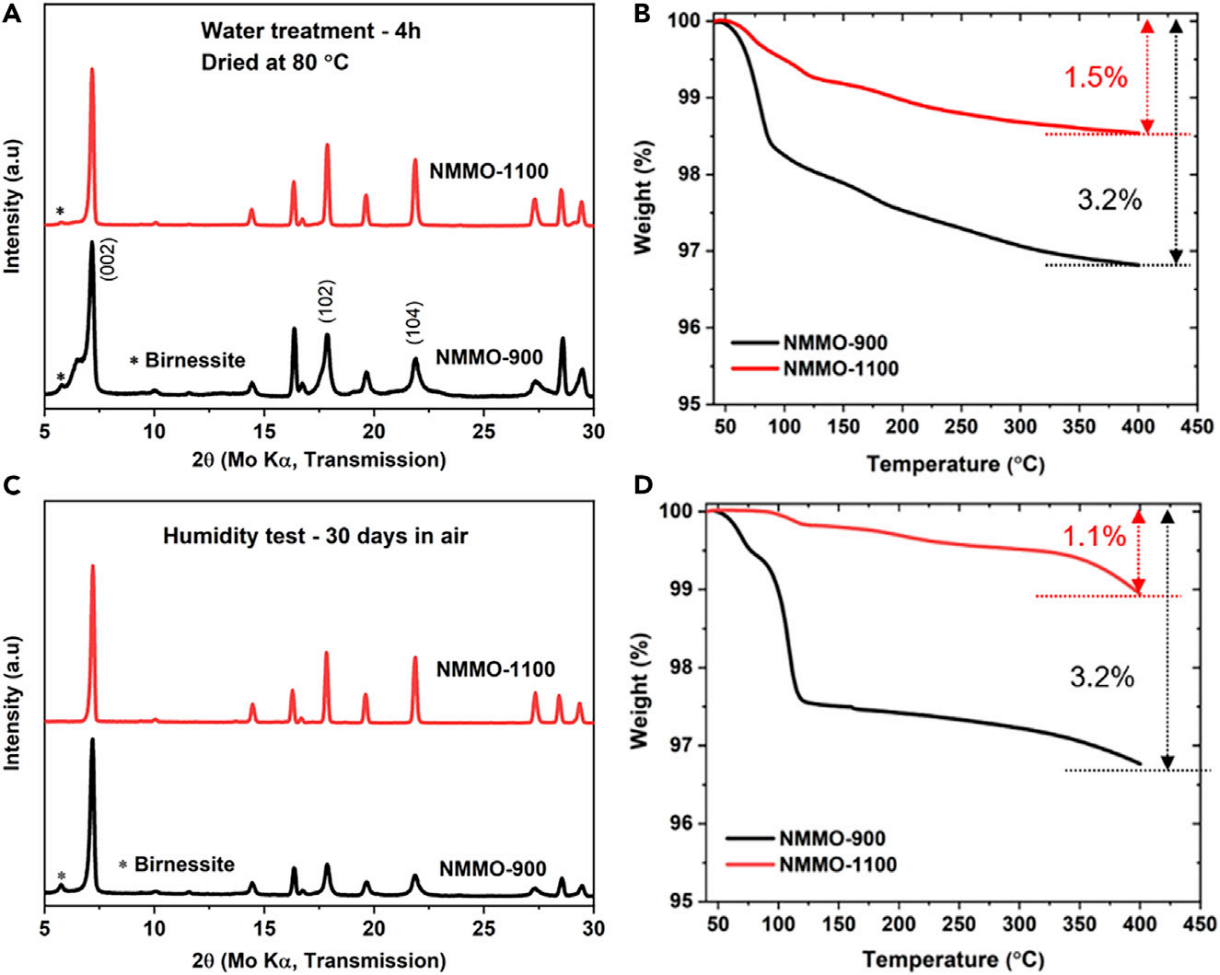
Phase analysis and chemical stability studies on NMMO cathode materials (A–D) (A) XRD patterns recorded on NMMO-900 and NMMO-1100 after water treatment, (B) corresponding TG of water-treated samples. (C) XRD patterns recorded after exposing the samples to air for 30 days, (D) corresponding TG of air-exposed samples. See also Figure S4.

The reactivity of the layered oxide cathode materials with water is one of their drawbacks. NMMO heated at higher temperatures might be less reactive toward water due to the reduced surface area. To check this, NMMO-900 and NMMO-1100 powders were stirred in distilled water for 4 h and dried at 80°C. In another experiment, these samples were left in an ambient atmosphere for 30 days. XRD and thermogravimetry (TG) were recorded on all these samples to investigate any phase changes and water uptake (refer to Figure 2). Figure 2A and B show the XRD and TG profiles, respectively, of water-treated and dried samples. NMMO-900 has been affected adversely after water treatment. New shoulder peaks at lower angles were observed for 002, 102, and 104 planes, which could be due to the hydration of the Na-layer in NMMO. This hydration expands the MO_2_ layers and results in shoulder peaks. In addition, an extra peak was seen at 2θ = 5.79°. This peak could be indexed to Birnessite, which is considered as a highly hydrated phase of Na_0.7_MnO_2_ with a chemical formula of Na_x_TMO_2_. yH_2_O (Shan et al., 2019). Interestingly, water treatment has negligible effects on NMMO-1100. A very low intense Birnessite peak was observed.

NMMO-900 showed a sharp weight loss of 1.8% below 100°C, followed by a gradual weight loss between 100°C and 400°C, resulting in a total weight loss of 3.2% (Figure 2B). NMMO-1100 showed a weight loss of only 0.5% below 100°C and a total weight loss of 1.5%. The initial sharp weight loss could be due to the evaporation of adsorbed water, and the second weight loss could be due to the loss of hydrated water from the NMMO and Birnessite-type phase. Figure 2C and 2D show the XRD and TG, respectively, of samples left in the ambient atmosphere for 30 days. In this case also, NMMO-900 was significantly affected after exposure to air, but not to the extent of water-treated NMMO-900. No evidence was seen for the formation of hydrated NMMO, but the appearance of the peak at 5.79° confirms the formation of the Birnessite-type phase. NMMO-900 showed a sharp weight loss of 2.5% below 120°C with a total weight loss of 3.2%. Though the total weight loss was 3.2% in both water-treated and air-exposed NMMO-900, the higher amount of weight loss in the first step of air-exposed NMMO-900 reveals the higher amount of surface-absorbed water and weight loss because the Birnessite phase is low. XRD also reveals a relatively low amount of Birnessite phase in air-exposed NMMO-900. In turn, NMMO-1100 showed a total weight loss of 1.1%, and no evidence was found from XRD for the formation of the Birnessite phase. In any case, NMMO could be restored after heating the water-treated and air-exposed samples at 300°C for 2 h (Figure S4). The negligible reactivity of NMMO-1100 with water and air is an important feature of the MSSC cathode. Overall, our results indicate that NMMO-1100 can be used with aqueous binders like CMC—which were overlooked otherwise due to the chemical incompatibility of layered cathodes with water.

### Microstructure and particle sizes of NMMO

**Figure 3:**
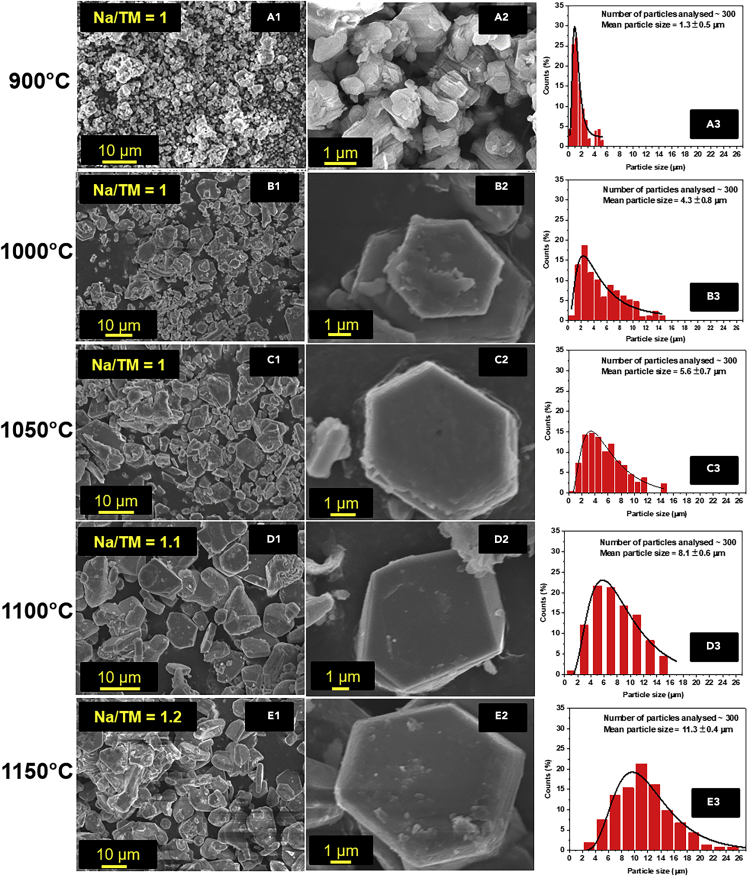
Microstructural analysis of NMMO cathode materials (A1–E3) Low-magnification SEM image, (A2) high-magnification image, (A3) particle size distribution of NMMO-900 sample. Similarly, (B1–B3) NMMO-1000, (C1–C3) NMMO-1050, (D1–D3) NMMO-1100, (E1–E3) NMMO-1150. Particle size was manually measured by measuring the longest dimension of 300 particles from several SEM images by using ImageJ software. See also Figures S5–S9.

**Figure 4:**
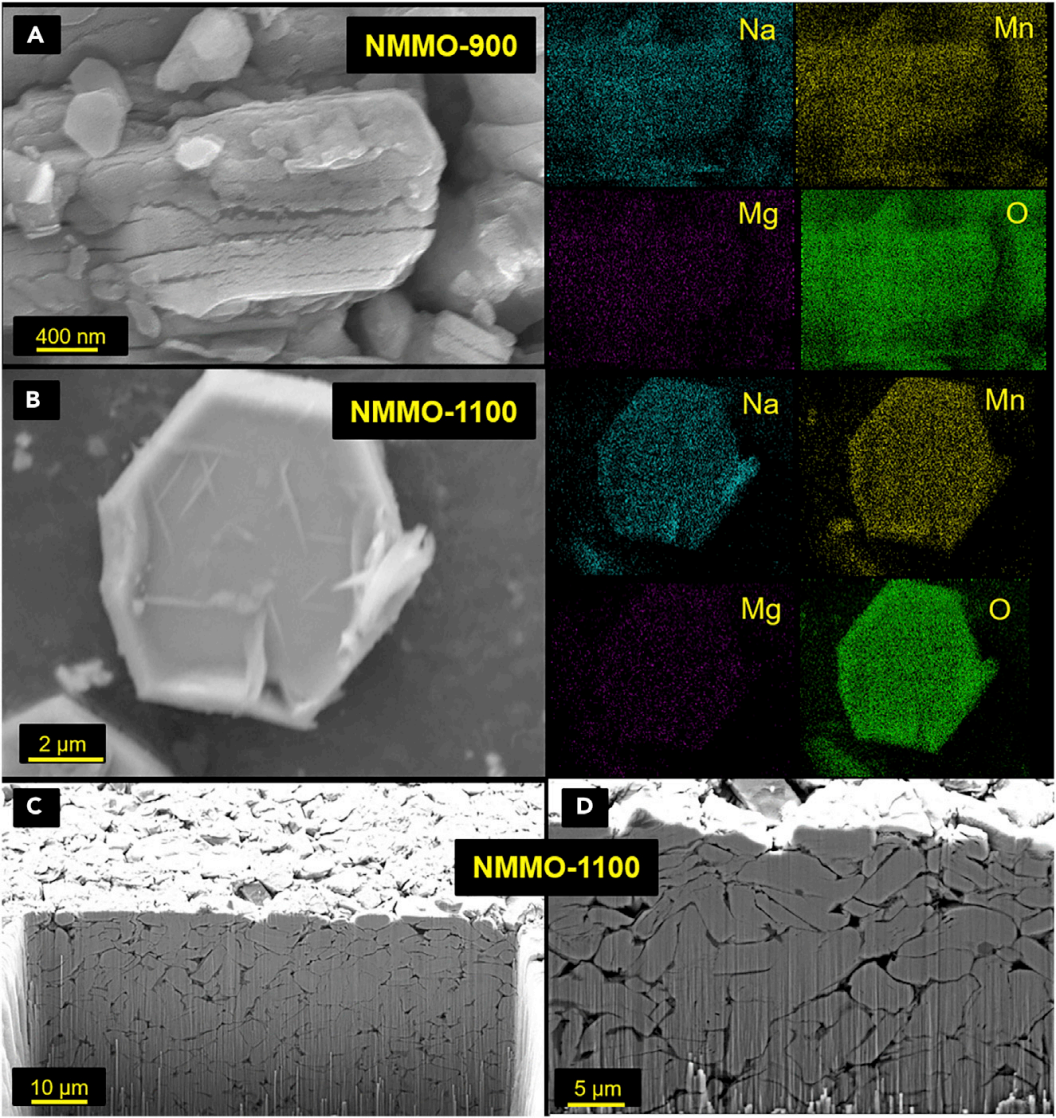
EDX and FIB analysis (A) SEM image of NMMO-900 and corresponding elemental mappings. (B) SEM image of NMMO-1100 and corresponding elemental mappings. (C and D) SEM images of NMMO-1100 pellet that were taken on focused ion-beam-milled area. See also Figures S10–S12.

Scanning electron microscopy (SEM) was performed on NMMO calcined at different temperatures to probe the particle shapes and size distribution. Figure 3 shows SEM images and primary particle size distribution of NMMO calcined at different temperatures. Figures 3A1, 3A2, 3B1, 3B2, 3C1, 3C2, 3D1, 3D2, 3E1, and 3E2 correspond to cathode materials calcined at 900°C, 1000°C, 1050°C, 1100°C, and 1150°C, respectively. The corresponding primary particle-size distribution plots are shown in Figures 3A3–3E3. The measured mean primary particle sizes (D_mean_) are 1.3 ± 0.5 μm, 4.3 ± 0.8 μm, 5.6 ± 0.7 μm, 8.1 ± 0.6 μm, and 11.3 ± 0.4 μm for samples calcined at 900°C, 1000°C, 1050°C, 1100°C, and 1150°C, respectively. NMMO-900 has highly irregular-shaped particles with more and large agglomerations. An increase in particle size and de-agglomeration was observed with the increase in calcination temperature. Further, particles developed smoother and crack-free surfaces with hexagonal platelet-like morphology with increasing calcination temperature. NMMO-900 exhibited narrow size distribution with most of the particles in the sub-micrometer range (<1 μm). In contrast, the remaining samples showed broad size distribution ranging from a sub-micrometer to 25 μm. Additional SEM images are provided in SI (Figures S5–S12) for each sample for further inspection. It may be important to note that the width of the particles was used to calculate the size distribution. This seems to be large, as the thickness of the hexagonal platelets may be significantly lower than the width. The average thickness of the hexagonal platelets of NMMO-1100 was 1.7 ± 0.47 μm (average of 60 particles). High-resolution SEM and EDX were performed to investigate the surface for any visible cracks. NMMO-900 particles exhibited rough surfaces with cracks (Figure 4A). In contrast, NMMO-1100 particles had a smoother surface without cracks (Figure 4B). This is also evident from the corresponding EDX elemental mappings showing uniform Na, Mn, and Mg elements distribution. On the other hand, NMMO-900 particles exhibited weak and diffused elemental mappings. SEM images of the NMMO-1100 exposing the *c axis* are given in the SI (Figure S9).

We also performed focused ion beam (FIB) milling on NMMO-1100 to investigate the particle cores. Here, NMMO-1100 powder was pressed into a pellet, FIB milling was performed on the pellet, and SEM images were taken. Figures 4C and 4D show the SEM images taken on FIB-milled area. As it is evident from the SEM images, the core of the particle of NMMO-1100 is also crack-free. Additional SEM images of the FIB-milled area are given in SI (Figure S11). Furthermore, small particles were visible on the surface of NMMO particles calcined at 1100°C and 1150°C. These particles could be surface impurities that evolved due to the excess use of Na_2_CO_3_ during the calcination of these samples. To check this, NMMO-1100 was washed with deionized water and heated at 300°C, and SEM images were recorded. The surface of these samples was much smoother compared to the untreated sample (Figure S12). From the above analysis, it is apparent that NMMO-900 is composed of polycrystalline particles, while NMMO-1100 and NMMO-1150 are composed of single-crystalline particles.

### Electrochemical performance and capacity degradation mechanism of NMMO

**Figure 5:**
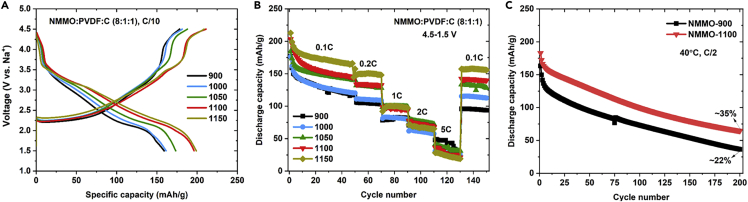
Electrochemical performance of cathode materials (A) Second charge-discharge profiles of all five NMMO cells obtained in the potential window of 4.5–1.5 V at a current rate of C/10. (B) Cycling performance of the same cells cycled at different current rates at 25°C. (C) Cycling performance of NMMO-900 and NMMO-1100 cycled at 40°C, C/2 rate.

The electrochemical performance of all NMMO cathodes was evaluated in half cells with Na metal as an anode. Figure 5 shows the electrochemical performance of all NMMO in the potential range of 4.5–1.5 V. The key results are summarized in Table 1. The electrochemical performance of several cathode materials reported in the literature for SIBs was tabulated and compared with NMMO (see Table S2). Figure 5A shows the second charge-discharge profiles of all five NMMO cells. The reversible capacities of NMMO-900, NMMO-1100, and NMMO-1150 cells were 159 mAh/g, 196 mAh/g, and 199 mAh/g, respectively. A reversible capacity of ∼160 mAh/g was reported for polycrystalline NMMO synthesized at 800°C (Billaud et al., 2014), which matches well with the polycrystalline NMMO-900. However, the capacity of the NMMO-900 cell faded significantly with cycling and reached a value of 94 mAh/g after 150 cycles corresponding to a capacity retention of 53% (Figure 5B). In contrast, higher capacity retention was observed for NMMO-1100 and NMMO-1150 cells. A reversible capacity of 140 mAh/g and 156 mAh/g was observed for NMMO-1100 and NMMO-1150 cells corresponding to capacity retention of 69% and 73%, respectively, after 150 cycles. The cycling stability of NMMO cathodes is proportional to the particle size, which can be rationalized based on the reduced contact area between the particle and electrolyte. This will reduce any parasitic side reactions with liquid electrolytes and improve the cycling stability of the electrodes. However, the C-rate tests yielded mixed results. NMMO-1100 and NMMO-1150 retained higher capacity up to 2C. However, at the 5C rate, NMMO-900 showed higher capacity than NMMO-1100 and NMMO-1150.

**Table 1:** Summary of the electrochemical performance data in the potential window 4.5–1.5 V

Sample	RC (mAh/g)			CD (%)		CR (%)
	0.1C	2C	5C	0.1C–2C	0.1C–5C	after 150 cycles
NMMO-900	159	70	43	46	62	53
NMMO-1000	161	61	28	53	78	64
NMMO-1050	173	76	35	49	76	69
NMMO-1100	196	72	26	53	83	69
NMMO-1150	199	67	24	61	86	73
	1^st^ DC (mAh/g)			CR (%) - after 50 cycles		
Potential (V)	4.7–1.5	4.5–1.5	4.2–1.5	4.7–1.5	4.5–1.5	4.2–1.5
NMMO-900	183	177	180	50	66	70
NMMO-1100	210	203	204	69	71	83

RC, reversible capacity; CD, capacity decay; CR, capacity retention; DC, discharge capacity.

See also Table S2.

Reducing the particle size reduces the average diffusion path length. Furthermore, particle cracking in polycrystalline samples will enable the penetration of liquid electrolytes and increase the contact area (Ruess et al., 2020; Trevisanello et al., 2021). This should enhance the C-rate capability. Another factor that affects the rate capability is the diffusion constant. Unfortunately, there is no well-established correlation between the particle size and the diffusion constant. However, the diffusion in LiFePO_4_ was found to increase when the particle size decreased (Malik et al., 2010). Also, polycrystalline LiNi_0.5_Co_0.2_Mn_0.3_O_2_ exhibited a higher diffusion coefficient than a single crystalline counterpart (Zhong et al., 2020).

The reduction in particle size and increase in diffusion constant should enable high rate capabilities for polycrystalline NMMO. However, NMMO-1100 was best up to 2C, and NMMO-900 was better at 5C. As the initial capacities were significantly different for NMMO-1100 and NMMO-900 (37 mAh/g higher for NMMO-1100), we compared the relative decay in capacity within the sample rather than comparing the capacities of different samples at different C-rates to better understand the trend. The % of capacity decay observed for NMMO-900 from 0.1C to 2 and 5C was 43% and 62%, respectively, whereas, it was 53% and 83% for NMMO-1100. Polycrystalline NMMO-900 showed relatively less decay in the capacity when the C-rate was increased. However, NMMO-1100 was better up to 2C when the real capacities were compared. Further, NMMO-1100 showed higher capacity retention of 35% after 200 cycles when cycled at 40°C. Whereas, NMMO-900 showed only 22% capacity retention (Figure 5C).

**Figure 6:**
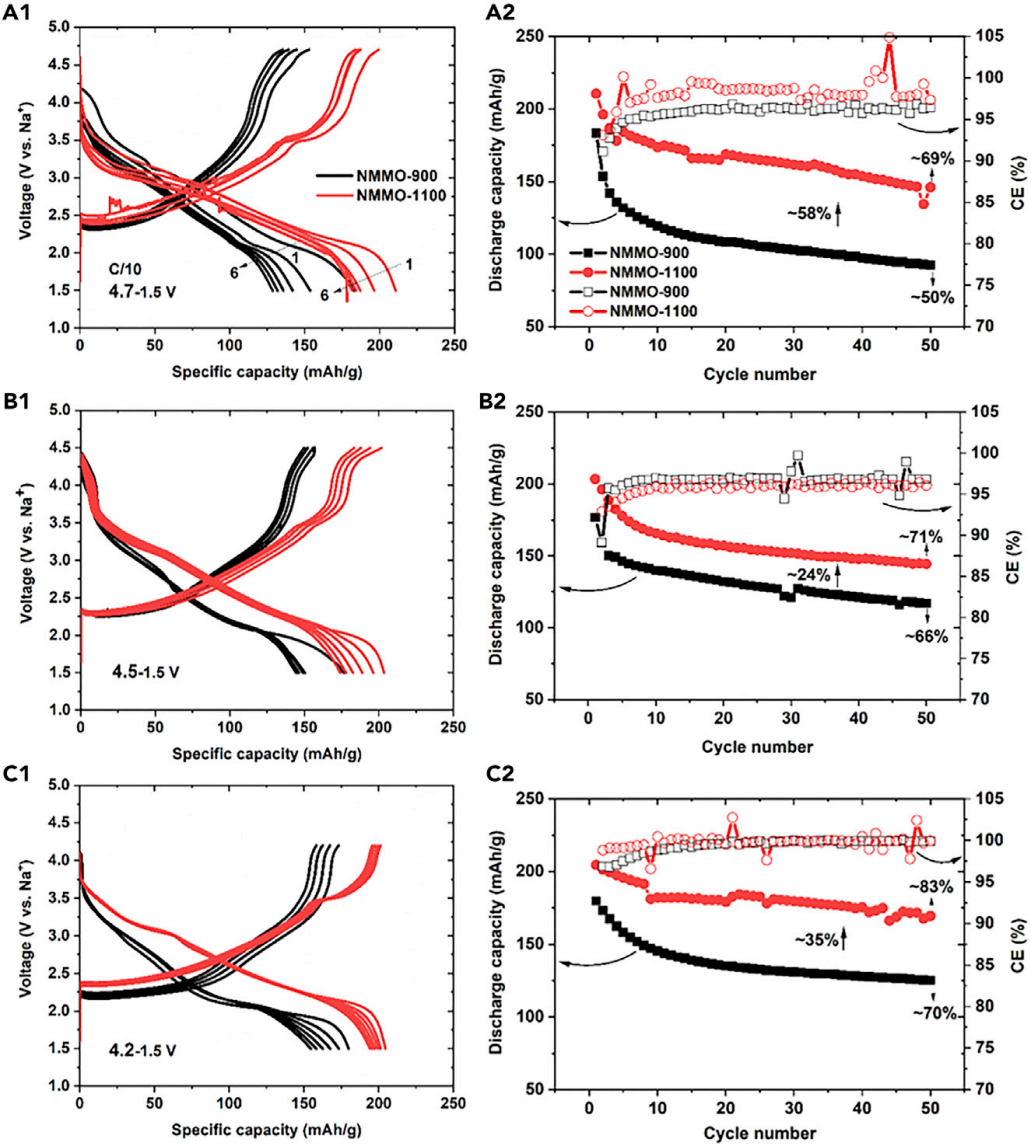
Electrochemical performance of NMMO-900 and NMMO-1100 in different potential windows under a constant current rate of C/10 (A1–C2) First discharge to sixth charge profiles obtained in the voltage window of 4.7–1.5 V, (A2) discharge capacity and coulombic efficiency (CE) of the cell as a function of cycle number: (B1) first discharge to sixth charge profiles obtained in the voltage window of 4.5–1.5 V, B2) discharge capacity and CE of the cell as a function of cycle number: (C1) first discharge to sixth charge profiles obtained in the voltage window of 4.2–1.5 V, (C2) discharge capacity and CE of the cell as a function of cycle number. See also Figure S13.

Figure 6 shows the charge-discharge profiles and cycling behavior of NMMO-900 and NMMO-1100 cells obtained in different potential windows at a C/10 rate. The first discharge capacity of NMMO-900 was increased to 183 mAh/g when charged up to 4.7 V (Figure 6A1). Electrolyte decomposition was observed when charged beyond 4.7 V (Figure S13). However, rapid capacity fading was observed in the potential window 4.7–1.5 V. Only 50% of its initial capacity was retained after 50 cycles. In contrast, the NMMO-1100 cell delivered 210 mAh/g in first discharge, and 69% of its initial capacity was retained after 50 cycles (Figure 6A2). By far, this is the best electrochemical performance for any layered cathode material with such a high upper cut-off voltage window for SIBs (Table S2). Higher capacity retention of 66% and 70% was observed for NMMO-900 cells when the charging voltage was limited to 4.5 and 4.2 V, respectively. In contrast, NMMO-1100 cells delivered an initial discharge capacity of 203 mAh/g and 204 mAh/g with 71% and 83% capacity retention after 50 cycles (Figure 6B2 and 6C2). Also, a sudden drop in the capacity was observed for NMMO-900 cells when they were cycled in the voltage window of 4.7–1.5 V and 4.5–1.5 V. The sudden capacity drop in the first cycle indicates an irreversible capacity loss (ICL). Overall, NMMO-1100 outperformed NMMO-900.

Though the single crystalline NMMO-1100 showed high cycling stability, its capacity retention was lower when compared with the results obtained for single-crystal cathodes of LIBs (Fan et al., 2020; Li et al., 2017). Therefore, more insight into the capacity fading mechanism is inevitable. Lei et al. investigated the thermodynamic phase stability of Na_x_CoO_2_ for SIBs with respect to Na content and found that P-type and O-type phases are stable between 0.68 < Na<0.76 and 0.83 < Na<1, respectively (Bianchini et al., 2020; Lei et al., 2014). Consequently, NMMO will undergo several reversible phase transformations between P-type and O-type during Na removal and reinsertion. During these phase transitions, the coordination of Na will switch between prismatic in P-type to octahedral in O-type co-ordinations. This will induce large volume changes between O-type and P-type phases. For example, a volume change of 15% was reported in Na_0.7_Mn_0.72_Mg_0.28_O_2_ when charged to 4.4 V (Yabuuchi et al., 2014b). Large volume changes will induce strain and cracking in particles, isolate particles from ionic and electronic transport, triggers parasitic side reactions, and eventually results in capacity fading. A comparison of the volume changes for SIB and LIB cathode materials was reported in Table S3. The volume changes reported for SIB cathode materials are in the range of 5%–30%. In contrast, the volume changes observed for LIB cathode materials were only 2% to 10%. The phase transitions in LIB-layered cathode materials are layered to spinel (O3 to O3) (Yahia et al., 2013). These transitions just require the movement of ions into the preformed empty sites. Therefore, volume changes are not high in this case. Another feature of layered cathode materials for SIB that is not common in LIB layered cathode materials is oxygen redox reactions at high charging voltages. The theoretical specific capacity of NMMO is 268 mAh/g, considering the removal and reinsertion of 1.0 Na/NMMO. Among these, 0.5 Na can be removed by oxidizing Mn^3+^ to Mn^4+^, corresponding to the capacity of 134 mAh/g. Removal of any extra Na should trigger oxygen redox or oxidize Mn^4+^ to Mn^5+^ or both simultaneously. However, it was shown that in Na_0.7_Mn_0.72_Mg_0.28_O_2_, oxidizing the host beyond Mn^4+^ would activate oxygen redox reactions rather than the formation of Mn^5+^ (Maitra et al., 2018). The oxygen redox enables high capacity but triggers oxygen release. It is known that Na_0.7_Mn_0.72_Mg_0.28_O_2_ would not release oxygen at RT due to the strong overlap of Mg p-orbitals with that of oxygen. However, oxygen may still be released in compounds like Na_0.7_Mn_0.9_Mg_0.1_O_2_, which are rich in transition metal. The high reversible capacity in NMMO also suggests that oxygen redox reactions are contributing significantly to its total reversible capacity. However, oxygen release might destabilize the structure gradually, induce particle cracking, and expose the new surfaces to the electrolyte. Independent from large volume changes, oxygen redox reactions could also contribute to the capacity fading.

**Figure 7:**
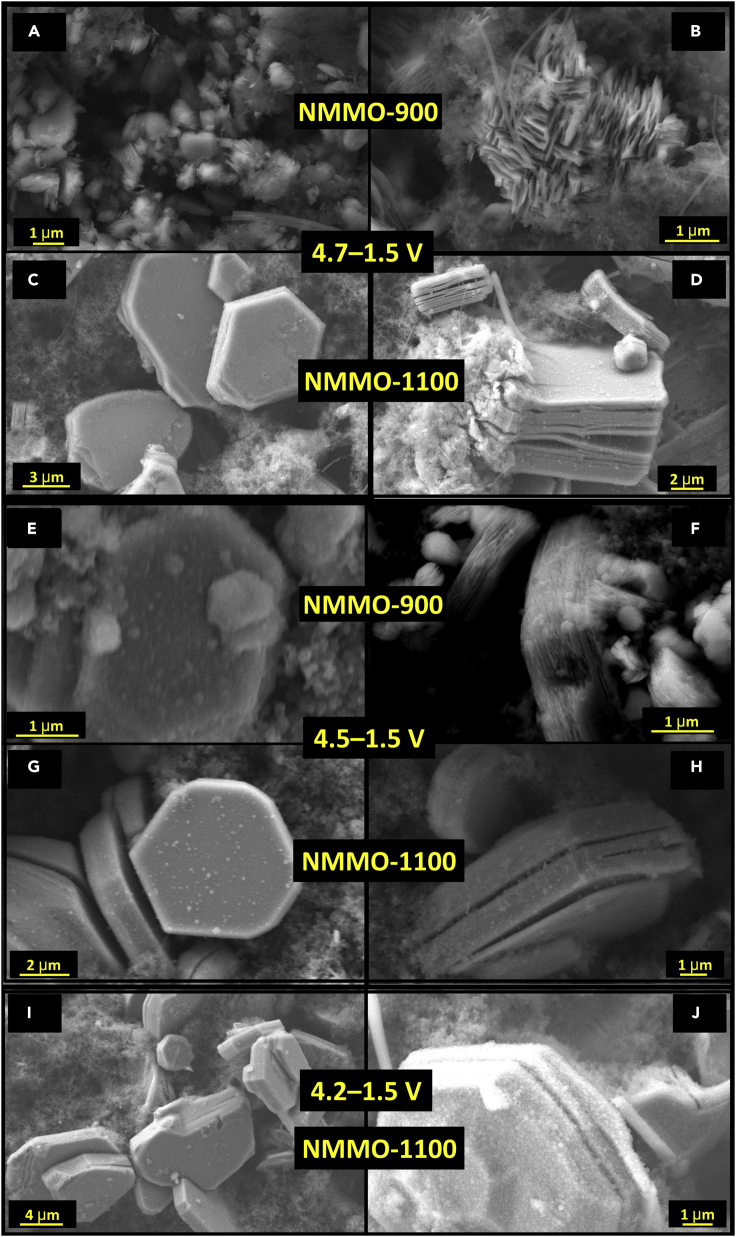
Microstructural analysis of cycled NMMO electrodes (A–J). SEM images of (A and B) NMMO-900 and (C and D) NMMO-1100-cycled electrodes in potential window 4.7–1.5 V. (E and F) and (G and H) SEM images of NMMO-900 and NMMO-1100-cycled electrodes, respectively, in the potential window 4.5–1.5 V. (I and J) SEM images of NMMO-1100-cycled electrode in the potential window 4.2–1.5 V. See also Figures S14 and S15.

We have investigated the microstructure of cycled NMMO electrodes to understand the impact of high volume changes, which eventually affecting its cycling stability. Figure 7 shows the SEM images of NMMO-900 and NMMO-1100 electrodes cycled in different voltage windows. Severe particle cracking was observed for the NMMO-900 particles after cycling between 4.7 and 1.5 V (Figures 7A and 7B). Large cracks were also seen in the NMMO-1100 particles after cycling between 4.7 and 1.5 V (Figure 7D). Relatively less cracking was observed when charging voltage was limited to 4.5 and 4.2 V. The cracking was seen mainly along the c axis, irrespective of the cycled voltage window. Hence, the phase transitions will mainly affect the particles along the c axis. Intra-particle cracking was reported for P2-type compounds due to the P to O-type transitions (Wang et al., 2018; Yang et al., 2020). Also, as can be seen from Figures S14 and S15, EDS mappings confirm that no segregation of elements was observed in both samples before and after cycling, respectively.

### Thermal behavior of charged NMMO cathodes

**Figure 8:**
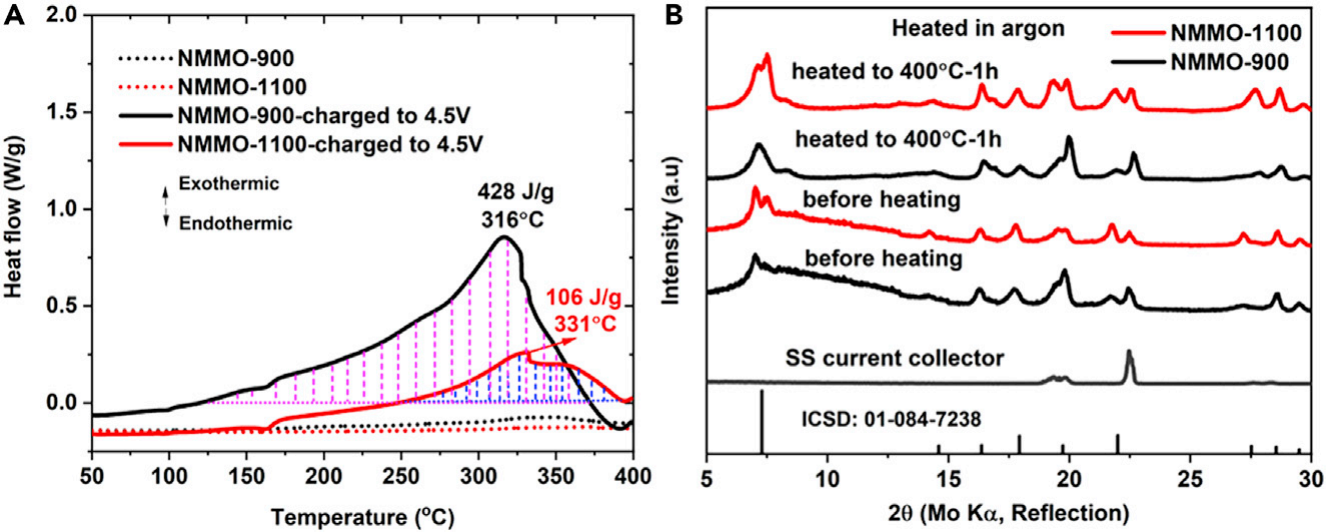
Thermal analysis of NMMO-900 and NMMO-1100-charged electrodes (A) DSC profiles recorded on charged electrodes (to 4.5 V) of NMMO-900, NMMO-1100, and the pristine samples. (B) *Ex situ* XRD patterns of NMMO-900 and NMMO-1100 taken on charged electrodes before and after heating them in Ar atmosphere at 400°C for 1 h.

One of the major concerns of layered cathode materials is their poor thermal stability in the charged state. To investigate the thermal stability of NMMO, DSC measurements were performed on charged cathodes of NMMO-900 and NMMO-1100. Figure 8 shows the thermal behavior of the charged (to 4.5 V) NMMO-900 and NMMO-1100 electrodes. NMMO-900 exhibited an exothermic peak at a temperature of 316°C with a total heat release of 428 J/g (Figure 8A). In contrast, the NMMO-1100 exhibited an exothermic peak at a temperature of 331°C with a total heat release of 106 J/g. The exothermic peak was shifted to higher temperatures by 15°C in NMMO-1100 and the heat released was 4 times lower than in the case of NMMO-900. This is a huge improvement in thermal stability. To understand the reasons for heat evolution and associated phase changes, *ex situ* XRD was performed on charged electrodes before and after heating at 400°C. The charged electrodes were washed with dimethyl carbonate (DMC) to remove any residual electrolyte or salt deposits on electrodes and dried at 80°C before DSC and XRD measurements. No major structural degradation was observed even after heating the electrodes at 400°C (Figure 8B). The heat release in layered compounds was attributed to oxygen release (Konarov et al., 2018, 2019b; Xie et al., 2018; Xu et al., 2017; Yu et al., 2015). The increased particle size and single-crystalline nature of the NMMO-1100 might have suppressed oxygen escape and improved thermal stability. The thermal stability of various cathode materials reported for SIB was shown in Table S4. Notably, NMMO-1100 is the best among reported compounds, with a minimum heat release of 106 J/g.

### Outlook

Micron-sized single-crystal cathodes have huge potential in electrochemical energy storage owing to their reduced surface area and electrode-electrolyte interactions. The advantage of single-crystal cathodes was well reported for LIBs. Inspired by the great advantages offered by MSSC in LIBs, we extended the concept to SIBs. Our results suggest that the advantages of MSSC observed in LIBs could not be merely extendable to SIBs, particularly the cycling stability. For example, though MSSC cathodes showed better cycling stability than the corresponding polycrystalline samples, the high cycling stability and capacity retention obtained for LIBs was not achieved with the single-crystal cathodes of SIBs. This could be due to the significant structural differences between SIB- and LIB-layered cathode materials. SIB-layered cathodes undergo huge volume changes (up to 23%) during cycling compared to 2% reported for LiNMC. These high volume changes are challenging to control, even in MSSC cathodes. Evidently, SEM images of cycled MSSC cathodes showed significant particle cracking. Though the particles are single crystals in nature initially, the particle cracking occurring during cycling will expose the internal surface of the particle to the electrolyte. This will lead to the decomposition of the electrolyte and eventually to capacity fading. Further, SIB-layered cathode materials show a high fraction of oxygen redox. This might gradually destabilize the structure if oxygen is released and also lead to capacity degradation. We suggest reducing the volume changes and oxygen release is vital to improve the cycling stability and capacity retention of MSSC cathodes for SIBs. Oxygen release can be mitigated by further doping. For example, Mg-rich Na_0.7_Mn_0.72_Mg_0.28_O_2_ does not show any oxygen release. Volume changes can be minimized by doping with suitable cations. Na_0.67_Ni_0.26_Mn_0.67_Zn_0.0.07_O_2_ shows the volume changes of only 7.25% when charged to 4.3 V. Further, the high volume changes and a fraction of oxygen redox can be minimized by lowering the charge voltage. For example, high capacity retention and less particle cracking were observed when NMMO electrodes were cycled between 4.2 and 1.5 V. However, the specific energy was reduced with lower cut-off voltages. The side effects due to particle cracking can be reduced by flexible surface coatings. By cumulatively implementing these strategies, sustainable and safe cathode materials can be developed for SIBs.

### Limitations of the study

We have not provided the direct proof of the single-crystal nature of the particle. Usually, particles without grain boundaries are called single crystals. To the best of our knowledge, only electron backscatter diffraction (EBSD) can confirm the nature of single crystals as it can spot the grain boundaries. Though selective area diffraction (SAED) can provide some insight, it will be confined to a very small area. Not suitable for submicron or micron-sized particles. Indeed, we extensively attempted to do EBSD, but our attempts were not fruitful due to the complexity of sample preparation. However, we have provided ample indirect evidence to prove the single-crystal nature of the particles. We have done HR-SEM to investigate the surface roughness of the particle, which did not show evidence for the grain boundaries on the surface. We have done the focused ion beam (FIB) technique to cut the particle and investigated the presence of any grain boundaries. We have performed BET analysis to probe the surface area and examine the presence of any pores, and this did not give evidence for the existence of any pores. Pores are expected if the grain boundaries are not unified.

Another limitation is we have not provided sufficient experimental evidence toward the poor rate capability of SC-NMMO cathodes. The cycling stability of SC-NMMO is proportional to the average size and better than PC-NMMO. The rate capability of SC-NMMO was not proportional to the average particle size, and the rate capability of PC-NMMO is better at the 5C rate. This behavior is not apparent at present. Detailed diffusion studies are required to understand this. Nevertheless, the rate capability of SC-NMMO is the interplay between diffusion pathlength, diffusion coefficient, and charge transfer resistance between electrolyte and cathode. Charge resistance is expected to be the same for all SC-NMMO samples as the surface structure and composition remain the same. However, diffusion path length and diffusion coefficient will change with respect to particle size, influencing the rate capability. Furthermore, the insight provided here might be limited to layered cathode materials and might not be extendable to other cathode materials.

## STAR★Methods

### Key resources table

**Table:** 

REAGENT or RESOURCE	SOURCE	IDENTIFIER
**Chemicals, Peptides, and Recombinant Proteins**		

Na_2_CO_3_	Alfa Aesar	CAS: 497-19-8
MnO_2_	Alfa Aesar	CAS:1313-13-9
MgO	Sigma-Aldrich	CAS: 1309-48-4
Carbon black	Alfa Aesar	CAS: 1333-86-4
Polyvinylidene fluoride (PVDF)	Alfa Aesar	CAS: 24937-79-9
N-Methyl-2-pyrrolidone (NMP)	Alfa Aesar	CAS: 872-50-4
Sodium metal	Sigma-Aldrich	CAS: 7440-23-5
NaClO_4_	Sigma-Aldrich	CAS: 7791-07-3
Propylene Carbonate	Sigma-Aldrich	CAS: 108-32-7
Fluoroethylene Carbonate	Sigma-Aldrich	CAS: 114435-02-8

### Resource availability

#### Lead contact

Further information and requests should be directed to the lead contact, M. Anji Reddy (a.r.munnangi@swansea.ac.uk).

#### Materials availability

Materials reported in this paper can be shared by the lead contact upon request.

### Method details

#### Synthesis and processing of the compounds

Reagents used for the synthesis of P2-Na_0.7_Mn_0.9_Mg_0.1_O_2_ (NMMO) are Na_2_CO_3_ (Alfa Aesar, purity 98%), MnO_2_ (Alfa Aesar, purity 99.9%), and MgO (Sigma-Aldrich, purity 97%). In a typical process, NMMO was synthesized by ball milling a stoichiometric amount of Na_2_CO_3_, MnO_2,_ and MgO for 2 h at 500 rpm and then heating at 900°C for 12 h in the air (named here as NMMO-900). This sample was again heated in different batches at 1000°C, 1050°C, 1100°C, and 1150°C for 12 h in air, denoted here as NMMO-1000, NMMO-1050, NMMO-1100, and NMMO-1150, respectively. The heating was performed in a box furnace with a 10°C/min rate followed by furnace cooling. Na/transition metal (TM) ratio was 1.1 and 1.2 for NMMO-1100 and NMMO-1150 samples, respectively. For the remaining three samples, Na/TM is 1.0. Extra Na was added to compensate for Na loss at high temperatures. For the water sensitivity test, few milligrams of each cathode material was stirred for 4 h in deionized water followed by centrifugation and drying at 80°C. For moisture-sensitivity test, few milligrams of each cathode material was spread on a Petri dish and kept in a fume hood for 30 days. Phase analysis was performed on the collected powders without further processing.

#### Physical characterization

The phase purity of the samples was analyzed by X-ray diffraction (XRD) measurements using an STOE -Stadi P diffractometer equipped with Mo K_α_ radiation source (λ = 0.709 Å) in transmission mode. Microstructural investigation of the powder samples, pristine electrodes, and cycled electrodes was performed using scanning electron microscopy (SEM, Crossbeam 340, ZEISS equipped with an EDX detector). To investigate the internal structure of NMMO-1100 active material powder, it was made into a pellet, and then the cross-sections of the pellet were prepared on a Capella FIB system using a Gallium ion source. The specific surface area of the samples was determined using Brunauer–Emmett–Teller (BET) method on a Micromeritics ASAP 2020 MP system.

#### Thermal analysis

Thermogravimetric analysis (TGA) (TA Discovery Instruments) was performed on pristine, water-treated, and moisture exposed samples. Differential scanning calorimetry (DSC) (TA Discovery Instruments) was performed on charged electrodes to quantify the amount of energy released during heating. Both TGA and DSC measurements were conducted from room temperature to 400°C under N_2_ atmosphere with a heating rate of 10 °C min^−1^. For DSC studies, electrodes were charged to an upper cut-off voltage of 4.5 V with a C/50 rate. The cells were then transferred to an Ar-filled glovebox and disassembled. The charged electrodes were washed/rinsed thoroughly with dimethyl carbonate (DMC) solvent to ensure complete removal of the electrolyte from the electrode. The washed electrodes were dried at 100°C for 2 h to remove the solvent. Then, the powder (∼2-3 mg) was scratched off the stainless steel (SS) current collector, filled in an aluminum crucible, and sealed before being taken out of the Argon-filled glovebox for the measurements.

#### Electrochemical testing

A uniform slurry was prepared using active material, carbon black, and PVDF in an 8:1:1 ratio with NMP as a solvent. This slurry was hand-coated on pre-cleaned SS current collectors (Φ-12 mm) with ∼ 2–3 mg mass loading. These electrodes were dried at 80°C for 12 h, followed by 120°C for 4 h. Electrochemical studies were performed in Swagelok-type half cells with sodium metal as the anode. Borosilicate glass fiber sheets (GF/C) were used as a separator. The electrolyte used was 1M NaClO_4_ in propylene carbonate (PC)/fluoroethylene carbonate (FEC) (98 + 2 v/v %). The cells were cycled at room temperature using an Arbin battery tester (BT-2000). The coatings and cell fabrication were performed in an Argon-filled glove box with a recirculation system (O_2_ and H_2_O < 1 ppm).

## Data Availability

Data reported in this paper can be shared by the lead contact upon request.

## Supporting citations

The following reference appears in the Supplemental Information: Bianchini et al., 2019; Choi and Manthiram, 2005; Deng et al., 2018; Dokko et al., 2000; Ghatak et al., 2018; Guo et al., 2015; Hamani et al., 2011; Hasa et al., 2014; Hou et al., 2016; Kaliyappan et al., 2018; Keller et al., 2016; Konarov et al., 2019a; Li et al., 2015, 2016, 2021a; Liu et al., 2016; Luo et al., 2016; Luo et al., 2017; Ma et al., 2011, 2017; Mao et al., 2019; Mu et al., 2015; Mullaliu et al., 2020; Nam et al., 2019; Tang et al., 2020; Van Der Ven et al., 2000; Vassilaras et al., 2013; Wang et al., 2015, 2016, 2019; Wu et al., 2016; Xiao et al., 2018; Xu et al., 2014; Yabuuchi et al., 2007, 2012b, 2014a; Yang et al., 2021; Yao et al., 2017; Yoshida et al., 2014; Zhang et al., 2016, 2019; Zhao et al., 2013; Zheng et al., 2016; Zhu et al., 2021.
