# Supplementary material for: Micron-sized single-crystal cathodes for sodium-ion batteries
Source: iScience. 2022 Apr 4;25(5):104205. doi: 10.1016/j.isci.2022.104205 (PMC9043968; doi:10.1016/j.isci.2022.104205)
Supplement: Document S1. Figures S1–S15 and Tables S1 — –S4 [file mmc1.pdf]

iScience, Volume *25*

Supplemental information

Micron-sized single-crystal cathodes for sodium-ion batteries

Venkat Pamidi, Shivam Trivedi, Santosh Behara, Maximilian Fichtner, and M. Anji Reddy

Supplementary Figures:

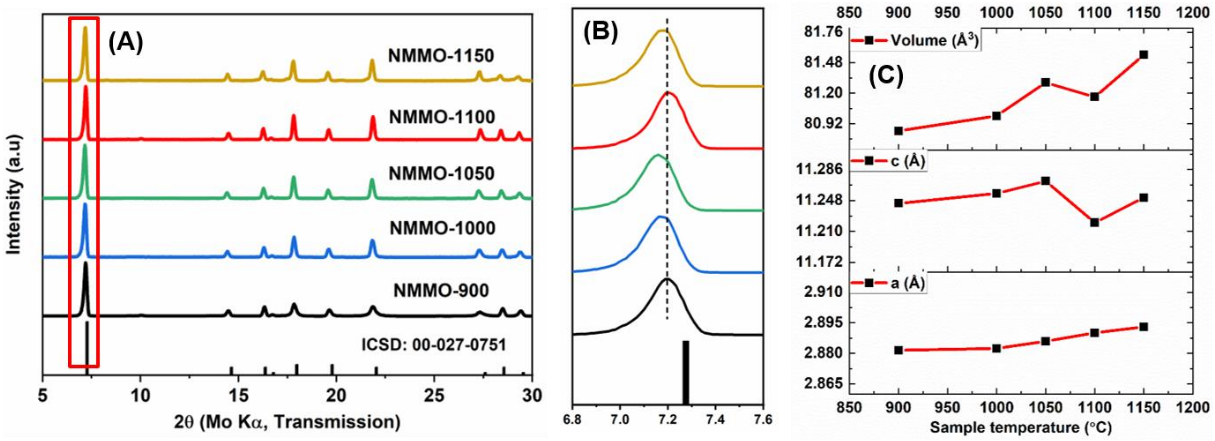

Figure S1. A) XRD patterns of NMMO synthesized at different temperatures. **B)** expanded view of (002) plane, highlighted with red color rectangular box. **C)** Lattice parameter evolution of NMMO heated at different temperatures. Related to Figure 1.

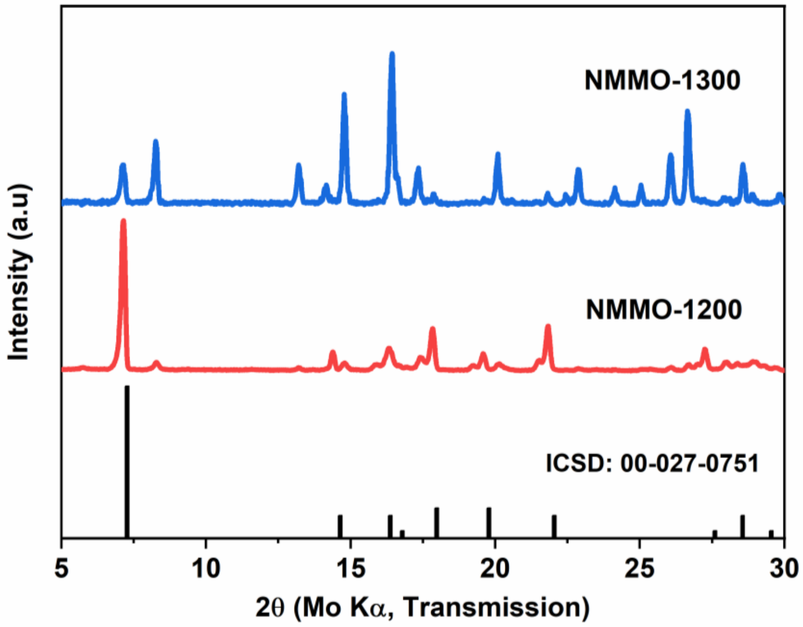

Figure S2. XRD patterns of NMO-900 when heated at 1200 °C and 1300 °C. Related to Figure 1.

Table S1. Refined parameters for $Na_{0.7}Mn_{0.9}Mg_{0.1}O_2$ at different temperatures. Related to Figure 1.

Material	$Na_{0.7}Mn_{0.9}Mg_{0.1}O_2$ (@900°C)	$Na_{0.7}Mn_{0.9}Mg_{0.1}O_2$ (@1000°C)	$Na_{0.7}Mn_{0.9}Mg_{0.1}O_2$ (@1050°C)	$Na_{0.7}Mn_{0.9}Mg_{0.1}O_2$ (@1100°C)	$Na_{0.7}Mn_{0.9}Mg_{0.1}O_2$ (@1150°C)
Lattice Parameter (Ǻ)	2.8815(7); 11.2444(4)	2.8824(6); 11.2567(9)	2.8859(2); 11.2717(2)	2.8901(1); 11.2206(4)	2.8930(0); 11.2515(6)
Volume (Å^3)	80.8544(9)	80.9935(1)	81.2985(1)	81.1656(8)	81.5526(2)
Atomic Positions					
Na1	2b	2b	2b	2b	2b
x	0.0000	0.0000	0.0000	0.0000	0.0000
y	0.0000	0.0000	0.0000	0.0000	0.0000
z	0.2500	0.2500	0.2500	0.2500	0.2500
Occupancy	0.0152(9)	0.0143(4)	0.0139(4)	0.0154(4)	0.0152(0)
Na2	2d	2d	2d	2d	2d
x	0.3333	0.3333	0.3333	0.3333	0.3333
y	0.6667	0.6667	0.6667	0.6667	0.6667
z	0.7500	0.7500	0.7500	0.7500	0.7500
Occupancy	0.0277(9)	0.0280(4)	0.0280(2)	0.0278(2)	0.0298(4)
Mn	2a	2a	2a	2a	2a
x	0.0000	0.0000	0.0000	0.0000	0.0000
y	0.0000	0.0000	0.0000	0.0000	0.0000
z	0.0000	0.0000	0.0000	0.0000	0.0000
Occupancy	0.0770(7)	0.0752(5)	0.0759(1)	0.0773(0)	0.0805(3)
Mg	2a	2a	2a	2a	2a
x	0.0000	0.0000	0.0000	0.0000	0.0000
y	0.0000	0.0000	0.0000	0.0000	0.0000
z	0.0000	0.0000	0.0000	0.0000	0.0000
Occupancy	0.0082(8)	0.0083(2)	0.0083(1)	0.0083(0)	0.0082(2)
O	4f	4f	4f	4f	4f
x	0.3333	0.3333	0.3333	0.3333	0.3333
y	0.6667	0.6667	0.6667	0.6667	0.6667
z	0.0901	0.0901	0.0901	0.0901	0.0901
Occupancy	0.1666(7)	0.1666(7)	0.1666(7)	0.1666(7)	0.1666(7)
χ^2	3.24	2.83	2.51	2.93	2.96

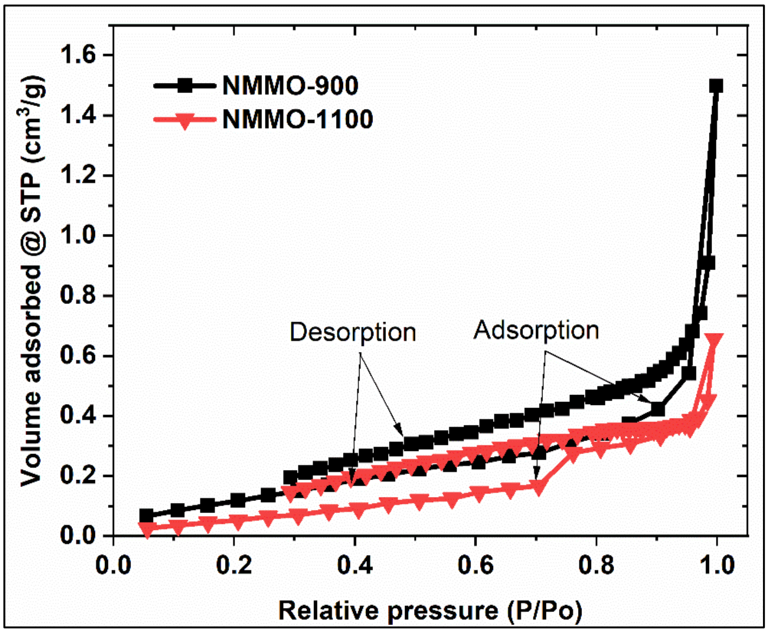

Figure S3. Nitrogen adsorption/desorption isotherms of NMMO-900 and NMMO-1100. Related to Figure 1.

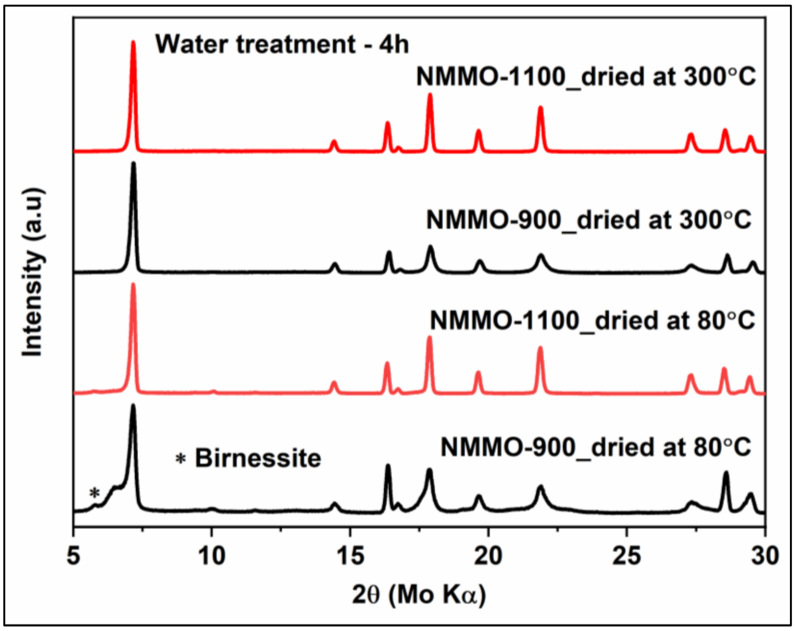

Figure S4. XRD patterns of water treated NMMO-900, NMMO-1100 and after drying them at 300 °C. Related to Figure 2.

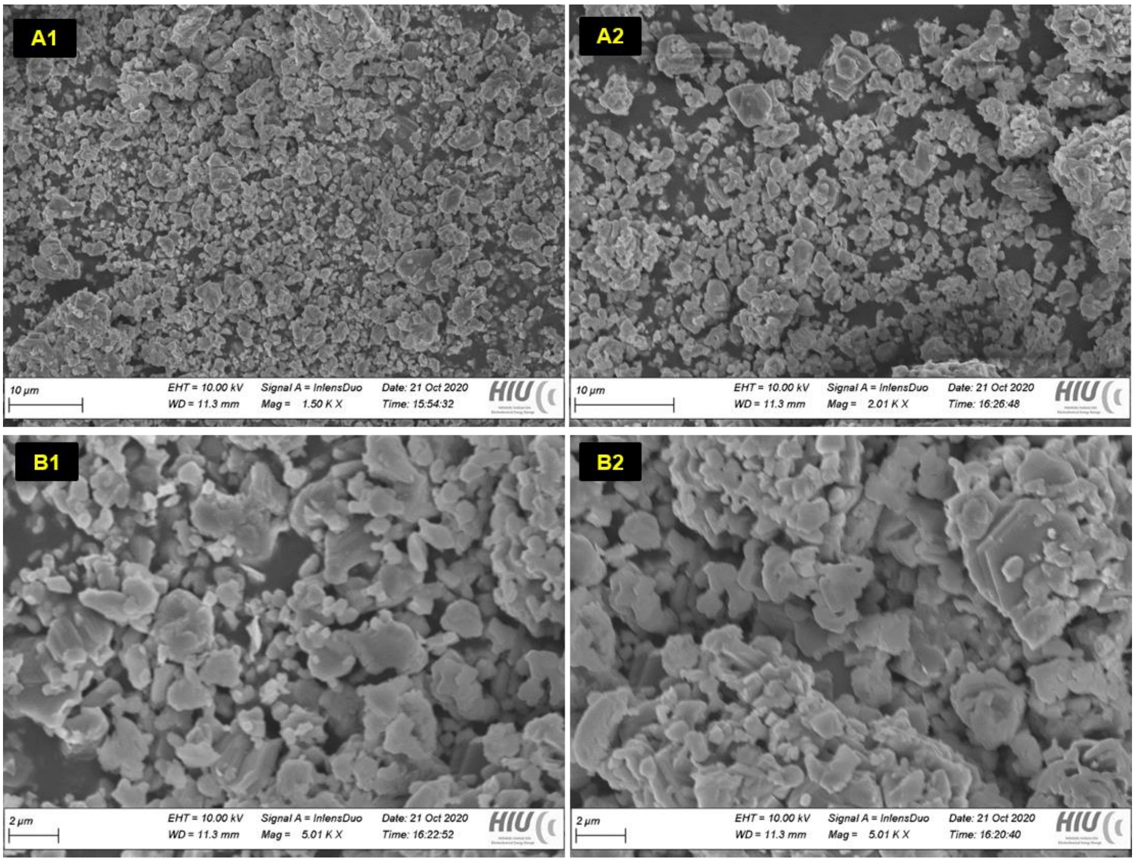

Figure S5. SEM images of NMMO-900 with different magnifications. Related to Figure 3.

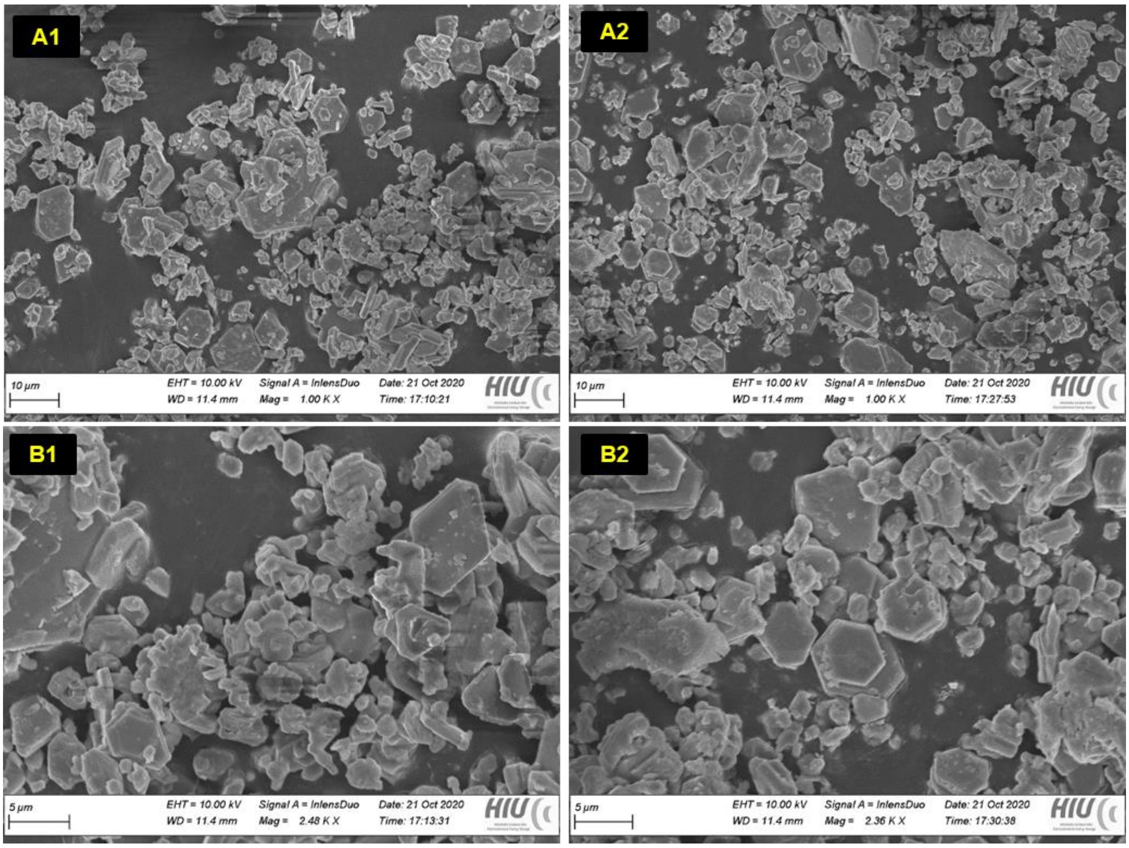

Figure S6. SEM images of NMMO-1000 with different magnifications. Related to Figure 3.

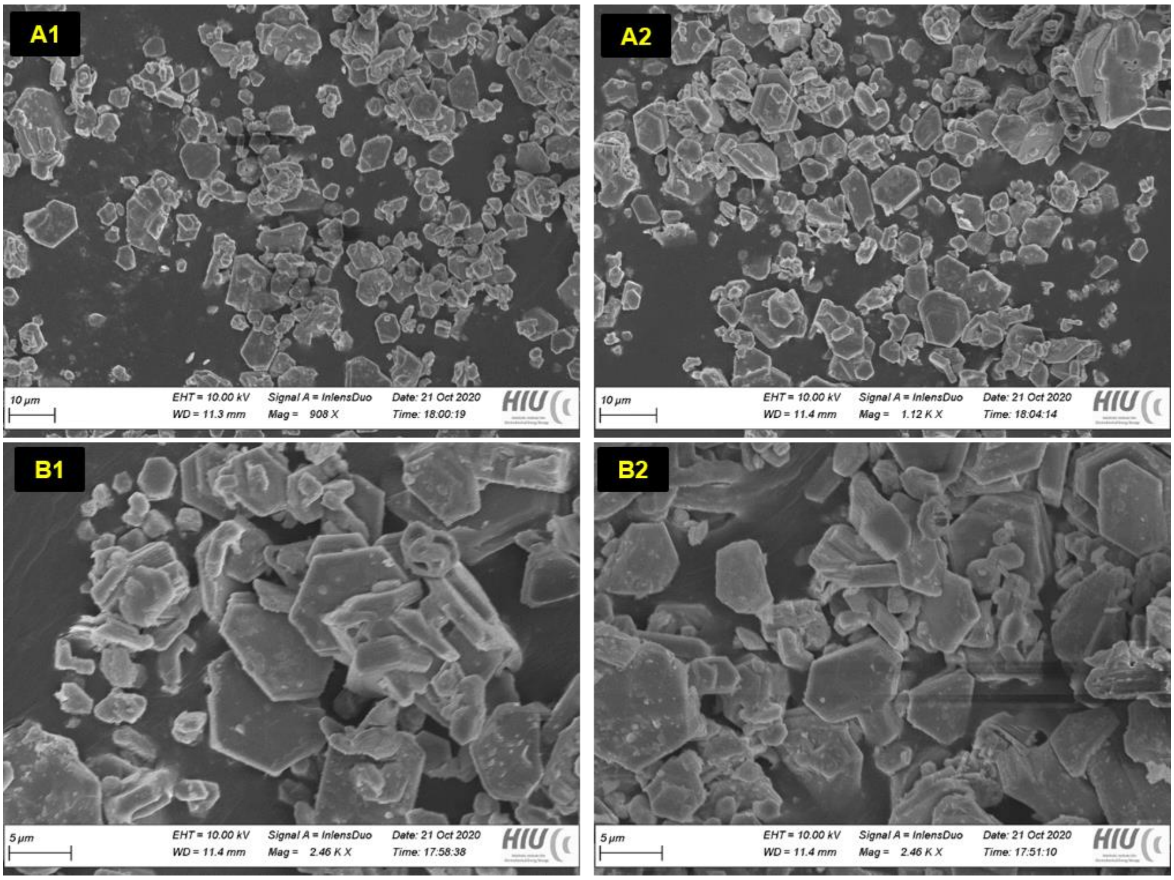

Figure S7. SEM images of NMMO-1050 with different magnifications. Related to Figure 3.

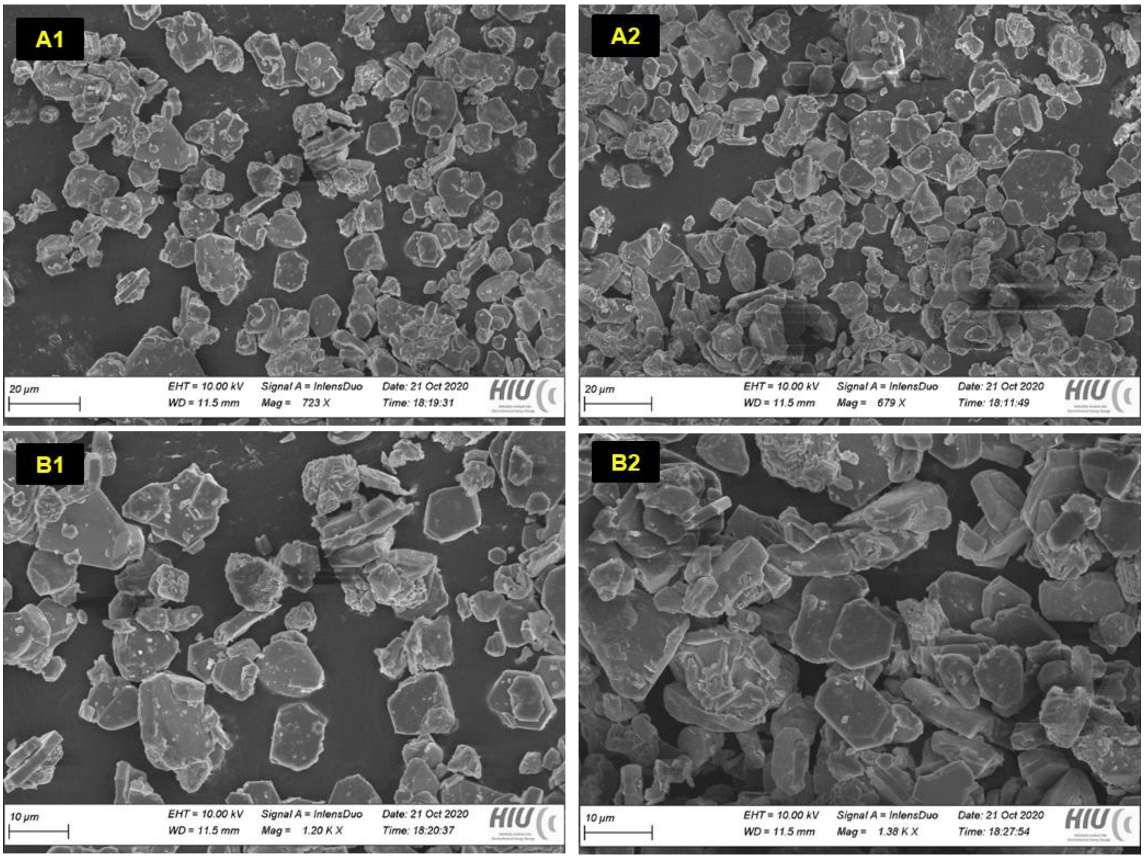

Figure S8. SEM images of NMMO-1100 with different magnifications. Related to Figure 3.

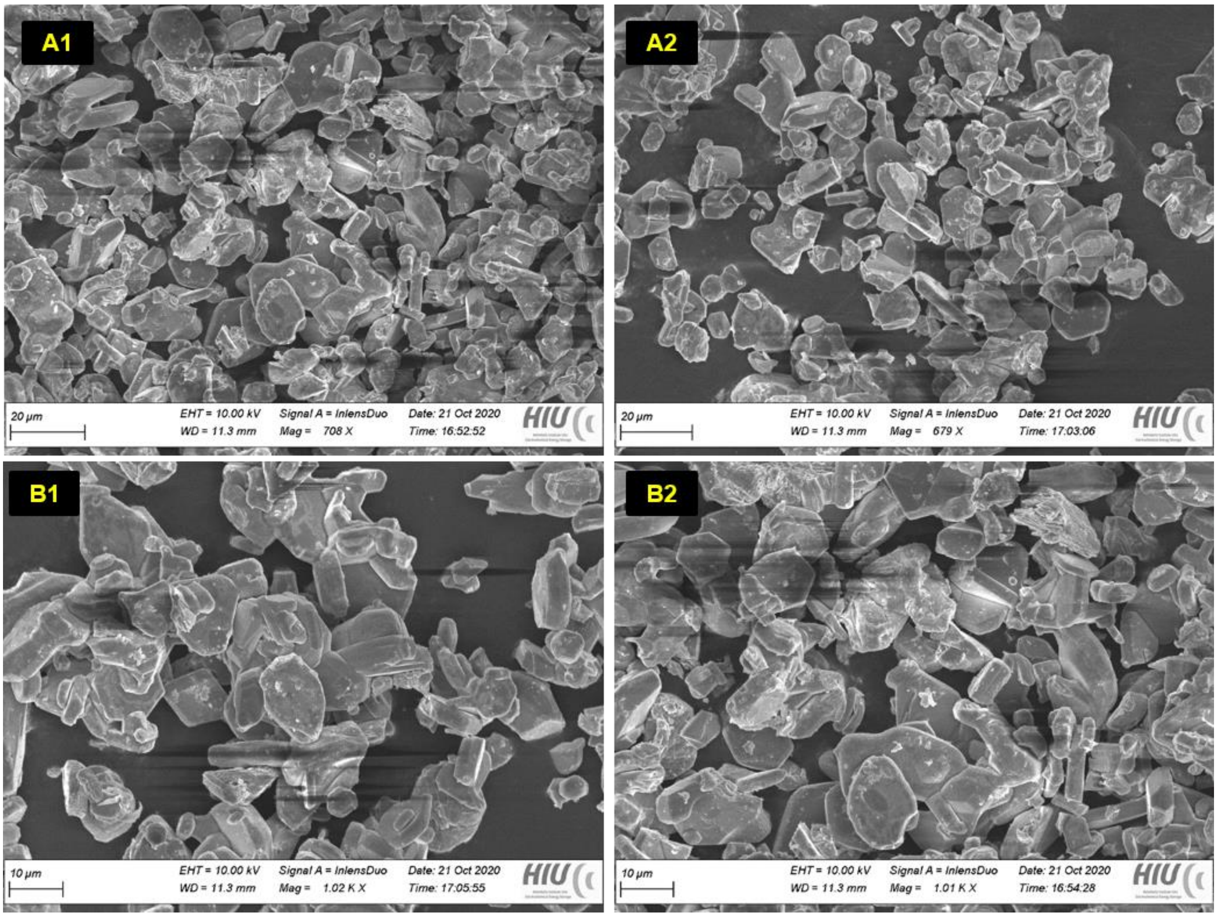

Figure S9. SEM images of NMMO-1150 with different magnifications. Related to Figure 3.

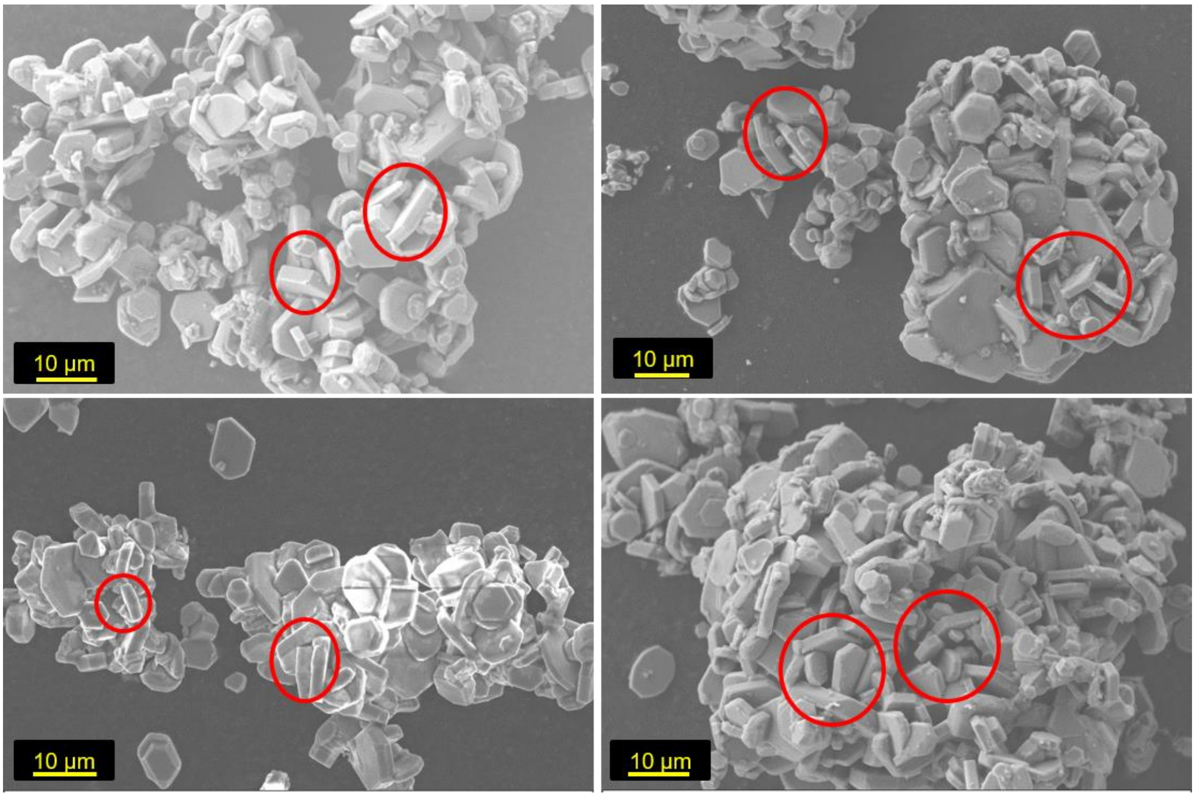

Figure S10. SEM images of NMMO-1100 sample highlighting the particles along c-axis. Related to Figure 4.

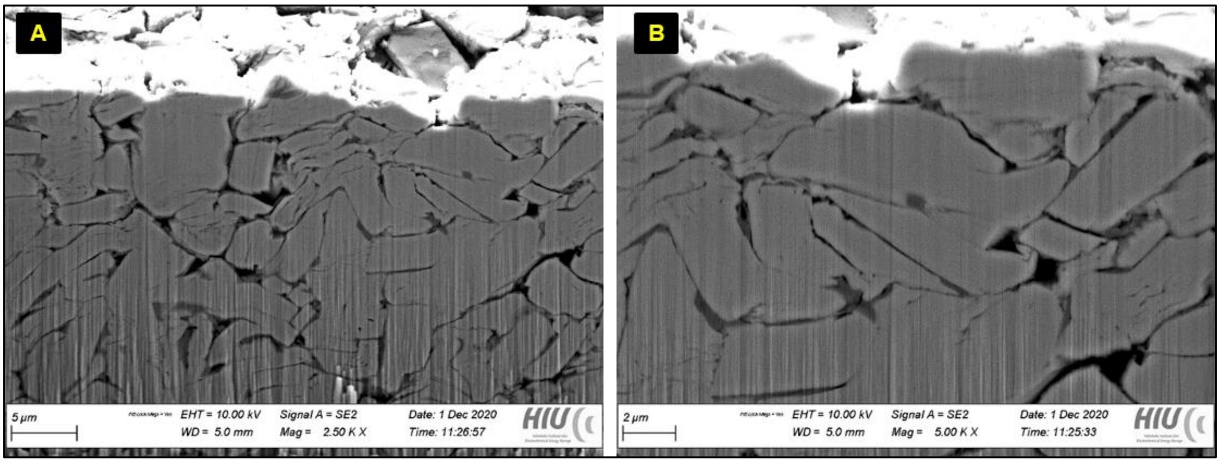

Figure S11. SEM images of NMMO-1100 sample taken on FIB milled area. Related to Figure 4.

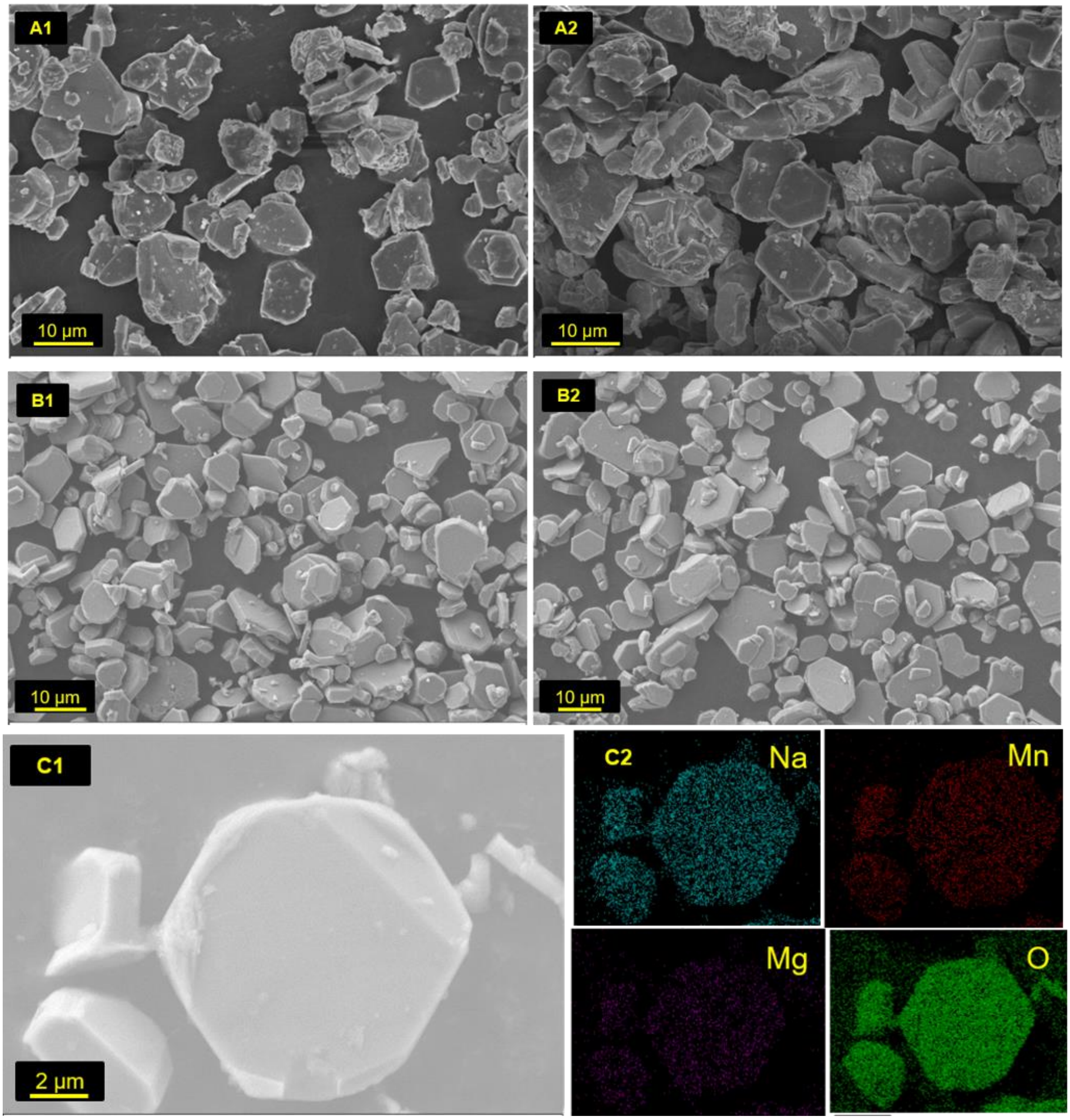

Figure S12. SEM images of as synthesised NMMO-1100 and NMMO-1100 after water treatment and drying at 300 °C. Related to Figure 4.

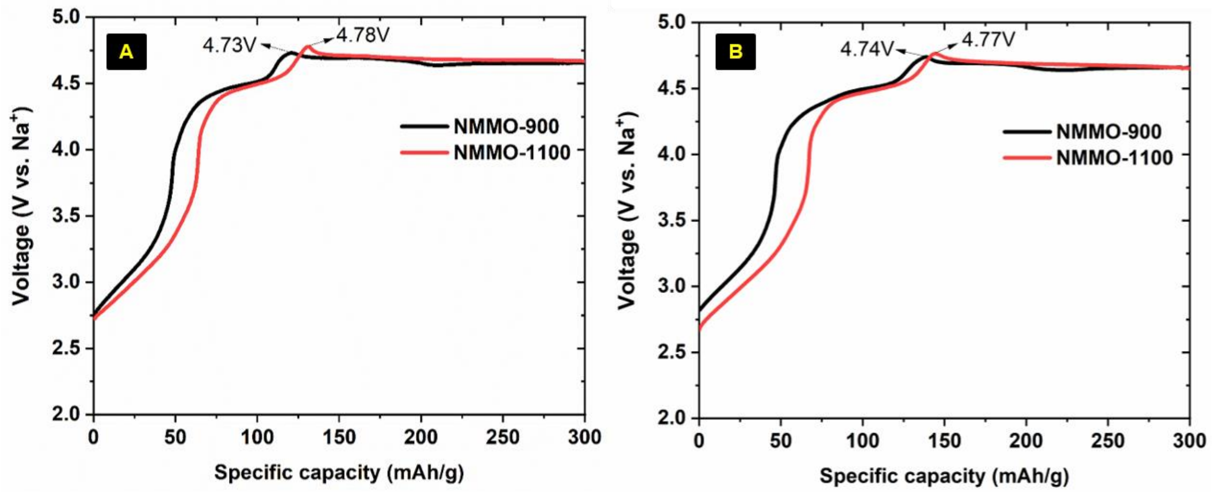

Figure S13. First charge profiles of NMMO-900 and NMMO-1100 when charged beyond 4.7V. Related to Figure 6.

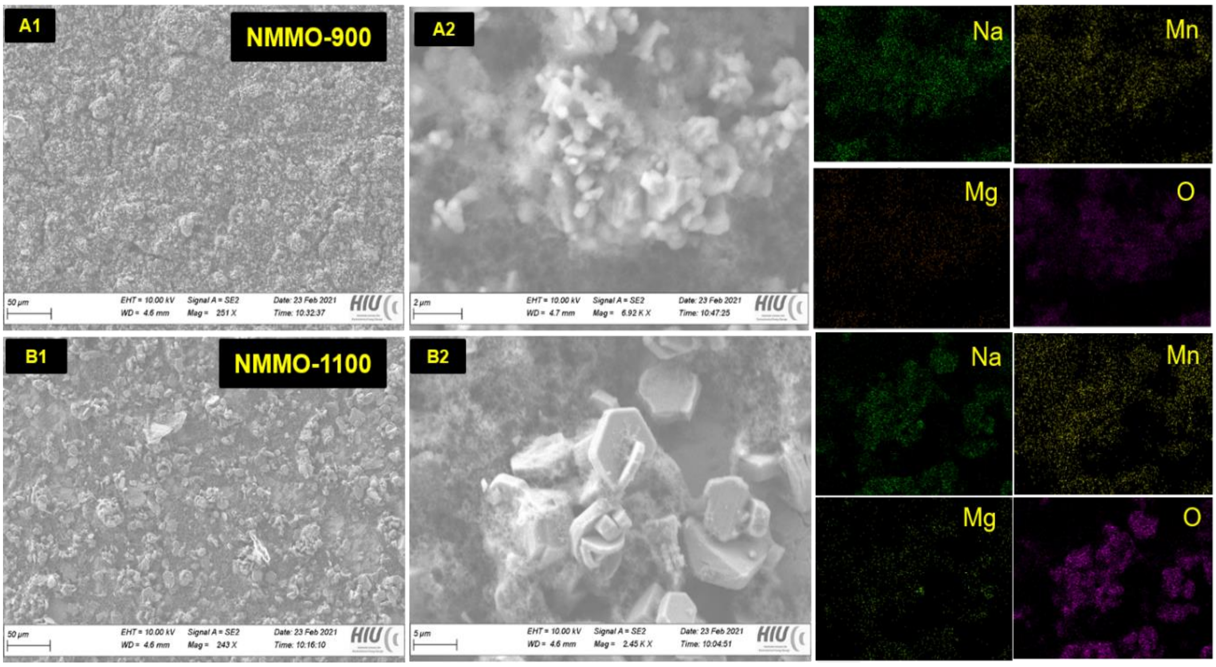

Figure S14. Microstructural analysis of pristine electrodes. (A1, A2) low and high magnification SEM images of NMO-900 pristine electrode, (B1, B2) low and high magnification SEM images of NMO-1100 pristine electrode. Their corresponding elemental mappings are given right side. Related to Figure 7.

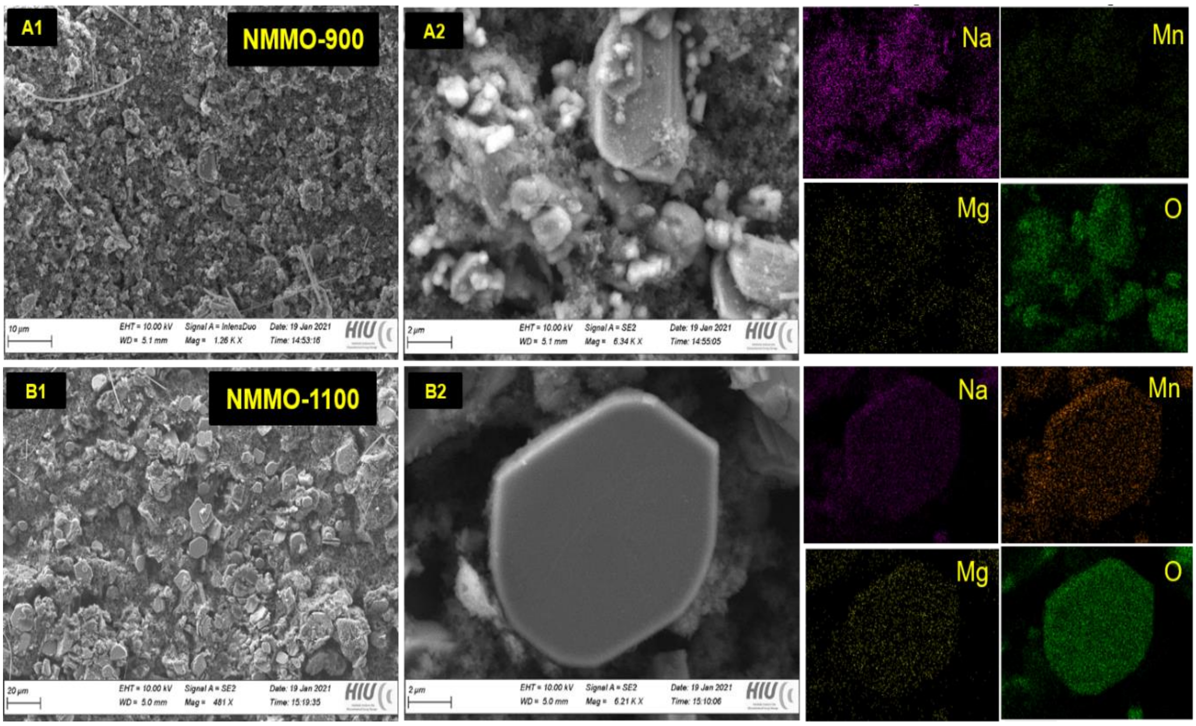

Figure S15. Microstructural analysis of cycled electrodes. (A1, A2) low and high magnification SEM images of NMO-900 cycled electrode, (B1, B2) low and high magnification SEM images of NMO-1100 cycled electrode. Their corresponding elemental mappings are given right side. Related to Figure 7.

Table S2. Summary of synthesis method, morphology and electrochemical performance of different layered oxide cathode materials. Synthesis method (SM): Solid-state (SS), Sol-gel (SG), Co-precipitation (CP), Thermal polymerization (TP), Hydrothermal (HT), Self-combustion reaction (SCR), Emulsion drying (ED), Ultrasonic spray pyrolysis (USP), Combustion (C), Liquid state (LS); Morphology (M): mean particle size (MPS), Primary particles (PP), Plates (P), Flakes (F), Rods (R); Microspheres (MS), Nanosheets (NS); Air stability (AS): Stable (S), Unstable (US); Discharge capacity (DC): Capacity retention (CR); Voltage window (VW); Cycle number (CN). Related to Figure 5 and 6.

Compound	SM	M (MPS), μm	AS	VW (V)	DC (mAh/g) /C-rate	CR (%) (CN)/ C-rate	Ref.
$NaMnO_2$	SS		US	2.0–3.8	185/0.1C	71(20)	(Ma et al., 2011)
$NaNiO_2$	SS		US	1.25–3.75	123/0.1C		(Vassilaras et al., 2013)
$NaFeO_2$	SS	P (0.5–1)	US	2.5–3.4	80/12 mA/g	75(30)	(Yabuuchi et al., 2012b)
$NaCrO_2$<br>C-$NaCrO_2$	ED	P	US<br>S	2.0–3.6	112/20mAh/g<br>121/20 Ah/g	80(50)<br>90(300)	(Yu et al., 2015)
$NaVO_2$<br>$Na_{0.7}VO_2$	SS		US	1.2–2.4	120/0.05C<br>100/0.05C		(Hamani et al., 2011)
$Na_{0.67}MnO_2$<br>$Na_{0.67}Ni_{0.33}Mn_{0.67}O_2$<br>$Na_{0.67}$Fe0.2$Ni_{0.15}Mn_{0.65}O_2$	USP	Slabs (~1)	-	2.0–3.8; 1.5–4.3	55;205/0.1C<br>105;158/0.1C<br>145;240/0.1C	70 (900)/1C	(Luo et al., 2017)
$NaNi_{0.5}Mn_{0.5}O_2$	SG	P (3–5)	US	2.0–4.0	141/0.05C	90(100)/0.05C	(Wang et al., 2016)
$Na_{0.67}Ni_{0.33}Mn_{0.67}O_2$<br>$Al_2O_3$- $Na_{0.67}Ni_{0.33}Mn_{0.67}O_2$	SS	P	S	2.5–4.3	~160/0.5C<br>~160/0.5C	~27(300)<br>~72(300)	(Liu et al., 2016)
$Na_{0.65}Ni_{0.25}Mn_{0.75}O_2$<br>$AlPO_4$-$Na_{0.65}Ni_{0.25}Mn_{0.75}O_2$<br>$Mg_3(PO_4)_2$-<br>$Na_{0.65}Ni_{0.25}Mn_{0.75}O_2$	CP	MS	US<br>S<br>S	1.5–3.75	135/0.2C<br>133/0.2C<br>130/0.2C	46(200)/1C<br>84(200)/1C<br>79(200)/1C	(Wang et al., 2019)
$Na_{0.67}Ni_{0.33}Mn_{0.67}O_2$	C	P (2.2)	S	2.3–4.1	87/0.1C	69(900)/1C	(Mao et al., 2019)
$Na_{0.78}Ni_{0.23}Mn_{0.69}O_2$	HT	P (3)	-	2.0–4.5	138/0.1C	90(50)	(Ma et al., 2017)
$Na_{0.67}Ni_{0.33}Mn_{0.67}Ti_{0.2}O_2$	SG	P (1–3)	S	2.5–4.3	113/0.2C	79(100)	(Tang et al., 2020)
$Na_{0.67}Ni_{0.33}Mn_{0.56}Al_{0.1}O_2$	LS	P (1–2)	-	1.6–4.0	138/0.1C	89(150)/1C	(Zhang et al., 2016)
$Na_{0.67}Ni_{0.23}Mn_{0.67}Mg_{0.1}O_2$	SG	NP	-	2.0–4.5	105/48mA/g	80(100)	(Hou et al., 2016)
$NaFe_{0.5}Mn_{0.5}O_2$<br>$Na_{0.67}Fe_{0.5}Mn_{0.5}O_2$	SS	PP (0.5)	US	1.5–4.3	~120/12mA/g<br>~190/12mA/g	62(30)<br>79(30)	(Yabuuchi et al., 2012a)
$Na_{0.9}Cu_{0.22}Fe_{0.3}Mn_{0.48}O_2$	SS	PP (3)	S	2.5–4.05	100/0.1C	97(100)	(Mu et al., 2015)
$Na_{0.78}Cu_{0.22}Fe_{0.11}Mn_{0.67}O_2$	SS	F (2–10)	S	2.5–4.2	89.0.1C	85(150)1C	(Li et al., 2015)

$Na_{0.83}Li_{0.25}Mn_{0.75}O_2$	SS	P (0.7–1.5)	US	1.5–4.4	190/10mA/g	92(20)	(Yabuuchi et al., 2014a)
$NaLi_{0.1}Ni_{0.35}Mn_{0.55}O_2$	CP	P (4–8)	US	2.0–4.2	128/12mA/g	85(100)	(Zheng et al., 2016)
$Na_{0.8}Li_{0.12}Ni_{0.22}Mn_{0.66}O_2$	CP	P	-	2.0–4.4	115/0.1C	91(50)	(Xu et al., 2014)
$Na_{0.67}Mn_{0.55}Ni_{0.25}Ti_{0.1}Li_{0.1}O_2$	SG	P (t-0.5)	-	1.5–4.2	130/0.1C	83(50)	(Li et al., 2016)
$Na_{0.8}Ni_{0.4}Ti_{0.6}O_2$	SS	PP (0.1–0.5)	US	2.0–4.0	83/0.2C	75(150)	(Guo et al., 2015)
$Na_{0.66}Ti_{0.34}Mn_{0.66}O_2$	SS	R (d-0.5, l-6)	S	2.5–3.9	~74/0.1C	88(400)	(Wang et al., 2015)
$Na_{0.67}Mg_{0.28}Mn_{0.72}O_2$	SS	P (1–3)	US	1.5–4.4	200/10mA/g	70(30)	(Yabuuchi et al., 2014b)
$Na_{0.67}Mg_xMn_{1-x}O_2$	SS or CP	P (3–4)	US	1.5–4.0	~175/12mA/g		(Billaud et al., 2014)
$Na_{0.67}Mg_{0.05}Mn_{0.95}O_2$	CP	-	US	1.5–4.0	170/100mA/g	91(100)	(Billaud et al., 2014)
$Na_{0.67}Mn_{0.7}Ni_{0.1}Fe_{0.1}Mg_{0.1}O_2$<br>$NaMn_{0.5}Ni_{0.3}Fe_{0.1}Mg_{0.1}O_2$<br>$Na_{0.76}Mn_{0.5}Ni_{0.3}Fe_{0.1}Mg_{0.1}O_2$	SS	F (1–4)<br>S (0.5–1)	S<br>US<br>US	2.0–4.3	134/0.1C<br>141/0.1C<br>153/0.1C	90(50)/1C<br>79(50)/1C<br>90(50)/1C	(Keller et al., 2016)
$NaNi_{0.45}Cu_{0.05}Mn_{0.4}Ti_{0.1}O_2$	SS	MP	S	2.0–4.0	124/0.1C	70(500)/1C	(Yao et al., 2017)
$NaLi_{0.05}Mn_{0.5}Ni_{0.3}Cu_{0.1}Mg_{0.05}O_2$	CP	MS	S	2.0–4.0	171/0.1C	63(1000)/5C	(Deng et al., 2018)
$NaLi_{0.05}Mn_{0.5}Ni_{0.3}Cu_{0.1}Mg_{0.05}O_2$	TP	NS (t-0.38)	-	2.0–4.0	130/0.5C	92(600)/5C	(Xiao et al., 2018)
$Na_{0.6}Ti_{0.2}Mn_{0.2}Co_{0.2}Ni_{0.2}Ru_{0.2}O_2$	SS	P (1–4)	US	1.5–4.5	164/0.1C	25(400)/0.2C	(Yang et al., 2021)
$Na_{0.66}Ni_{0.13}Mn_{0.54}Co_{0.13}O_2$	HT	MS	-	2.0–4.7	120/1C	90(150)	(Kaliyappan et al., 2018)
$Na_{0.5}Ni_{0.23}Fe_{0.13}Mn_{0.63}O_2$	CP	P (0.5)	US	1.5–4.6	150/0.5C	71(100)	(Hasa et al., 2014)
$Na_{0.6}Li_{0.2}Ni_{0.2}Mn_{0.6}O2$	SCR	P (4–5)	US	2.0–4.6	170/12mA/g		(De La Llave et al., 2016)
$Na_{0.67}Zn_{0.3}Mn_{0.7}O_2$	C	-	US	1.5–4.6	190/0.1C	80(200)	(Konarov et al., 2019a)
$Na_{0.7}Mn_{0.9}Mg_{0.1}O_2$	SS	P (1–10)	S	1.5–4.7<br>1.5–4.5<br>1.5–4.2	210/0.1C<br>203/0.1C<br>204/0.1C	69 (50)<br>69 (150)<br>83 (50)	**This work**

Table S3. Volume changes (VC) reported for some of the SIB and LIB layered cathode materials: Charging voltage (CV). Related to Figure 7.

Compound	VC (%)	CV (V)	Ref.
**SIBs**			
$Na_{0.67}Ni_{0.33}Mn_{0.67}O_2$	23.1	4.5	(Yoshida et al., 2014)
$Na_{0.67}Ni_{0.33}Mn_{0.5}Ti_{0.17}O_2$	12.7	4.5	
$Na_{0.67}Ni_{0.33}Mn_{0.33}Ti_{0.33}O_2$	12.1	4.5	
$Na_{0.67}Mg_{0.28}Mn_{0.72}O_2$	15	4.4	(Yabuuchi et al., 2014b)
$Na_{0.67}Ni_{0.33}Mn_{0.67}O_2$	30		(Lu and Dahn, 2001)
$Na_{0.67}Fe_{0.5}Mn_{0.5}O_2$	13	4.3	(Yabuuchi et al., 2012a)
$Na_{0.67}Ni_{0.26}Mn_{0.67}Zn_{0.0.07}O_2$	7.25	4.3	(Wu et al., 2016)
P/O $Na_{0.76}Mn_{0.5}Ni_{0.3}Fe_{0.1}Mg_{0.1}O_2$	11.9 (CCCV)<br>4.9 (CC)	4.3	(Mullaliu et al., 2020)
$Na_{0.67}Ni_{0.2}Mn_{0.8}O_2$	13	4.3	(Konarov et al., 2018)
**LIBs**			
$Li_{1-x}Co_{1/3}Ni_{1/3}Mn_{1/3}O_2$ (2/3<x<1)	5	5	(Yabuuchi et al., 2007)
$Li[Ni0.8Co0.16Al0.04]O_2$<br>$Li[Ni0.88Co0.10Al0.02]O_2$<br>$Li[Ni0.95Co0.04Al0.01]O_2$	5.63<br>7.10<br>8.37	4.3	(Nam et al., 2019)
$LiCoO_2$	4.27	4.2	(Luo et al., 2016)
$Li_{24}Ni_{22}Co_1Al_1O_{48}$<br>$Li_{22}Na_2Ni_{22}Co_1Al_1O_{48}$	2.78<br>6.08	~4	(Ghatak et al., 2018)
$Li_{1-x}Ni_{1/3}Mn_{1/3}Co_{1/3}O_2$ (1-x=0.2)	<2		(Choi and Manthiram, 2005)
$LiNiO_2$	9	4.2	(Dokko et al., 2000)
$LiNiO_2$	10		(Bianchini et al., 2019)
Spinel-layered $LiMnO_2$	3.26	4.5	(Zhu et al., 2021)
$Li_xMn_2O_4$ (x = 1 and 2)	5.6		(Van Der Ven et al., 2000)
$LiNi_{0.6}Co_{0.2}Mn_{0.2}O_2$	2.12	4.4	(Li et al., 2021)

Table S4. Thermal stabilities reported for some of the SIB cathode materials. Related to Figure 8.

Compound name	Exothermic peak temperature (°C)	Energy released	Charge voltage (V)	Ref.
$Na_{0.58}FeO_2$	360	0.18 J		(Zhao et al., 2013)
$NaCrO_2$<br>C-$NaCrO_2$	270–360<br>170–320		Chemical de-sodiation	(Yu et al., 2015)
$Na_xNi_{0.33}Mn_{0.33}Co_{0.33}O_2$ (deep charged)	260	120.6 J/g	4.4	(Xu et al., 2017)
$Na_{0.83}Li_{0.25}Mn_{0.75}O_2$<br>Zn-doped $Na_{0.83}Li_{0.25}Mn_{0.75}O_2$	~310<br>~360			(Zhang et al., 2019)
P2-$Na_{0.67}MnO_2$<br>P2-$Na_{0.29}Mn_{0.8}Ni_{0.2}O_2$	220.8<br>294.1		4.3	(Konarov et al., 2018)
P2-$Na_{0.29}Mn_{0.8}Co_{0.2}O_2$	265.6		4.6	(Konarov et al., 2019b)
NMMO-900<br>NMMO-1100	316<br>331	428 J/g<br>106 J/g	4.5	**This work**
